# Inequitable paediatric kidney transplantation in resource-limited countries: expert recommendations for Nigeria – a scoping review

**DOI:** 10.1136/bmjgh-2024-017023

**Published:** 2025-12-05

**Authors:** Emmanuel Ademola Anigilaje, Louise Oni, Olanrewaju Timothy Adedoyin, Felicia U Eke, Henrietta Okafor, Odutola Israel Odetunde, Paul Kehinde Ibitoye, Adebowale D Ademola, Michael Abel Alao, Oluseyi Oniyangi, Patience Ngozi Obiagwu, Adewale E Adetunji, Bidemi Adebukola Ajite, Adeline O Adaje, Ajayi Ajetomobi, Femi Mark, Datonye Christopher Briggs, Nonso Epundu

**Affiliations:** 1 University of Abuja College of Health Sciences, Abuja, Nigeria; 2 Department of Women’s and Children’s Health, University of Liverpool, Liverpool, UK; 3 Department of Paediatrics, University of Ilorin Teaching Hospital, Ilorin, Nigeria; 4 University of Port Harcourt Teaching Hospital, Port Harcourt, Rivers State, Nigeria; 5 Department of Paediatrics, University of Nigeria, Nsukka, Enugu State, Nigeria; 6 Department of Paediatrics, Usmanu Danfodiyo University, Sokoto, Sokoto, Nigeria; 7 Department of Paediatrics, University of Ibadan, Ibadan, Oyo State, Nigeria; 8 Department of Paediatrics, National Hospital, Calicut, India; 9 Department of Paediatrics, Aminu Kano Teaching Hospital, Kano, Kano State, Nigeria; 10 Department of Paediatrics, Irrua Specialist Teaching Hospital, Irrua, Edo State, Nigeria; 11 Department of Paediatrics, Ekiti State University Teaching Hospital, Ado Ekiti, Ekiti State, Nigeria; 12 Department of Paediatrics, Federal Teaching Hospital Ido-Ekiti, Ido Ekiti, Nigeria; 13 Department of Paediatrics, Federal Teaching Hospital, Lokoja, Lokoja, Kogi, Nigeria; 14 Department of Paediatrics, Rivers State University Teaching Hospital, Port Harcourt, Nigeria; 15 Department of Paediatrics, Zenith Medical and Kidney Centre, Abuja, Abuja, Nigeria

**Keywords:** Child health, Public Health, Global Health, Health services research, Review

## Abstract

**Background:**

In Nigeria and other resource-limited countries (RLCs), paediatric kidney transplantation (PKT) uptake remains low, causing preventable deaths from kidney failure.

**Objectives:**

This study identifies challenges and solutions for inequitable kidney transplantation (KT) in RLCs through a scoping review. Based on an analysis of questionnaires administered to Nigerian paediatric nephrologists (NPNs), consensus recommendations for expanding PKT in Nigeria are derived.

**Eligibility criteria:**

The Population-Intervention-Comparison-Outcome framework extracts data on the Population (challenges beleaguering adult KT or PKT in RLCs), Intervention (solutions offered for KT/PKT) and Comparison/Outcome (successes of solutions) of published articles.

**Sources of evidence:**

Literature published in the English language from January 2003 to December 2023 was sourced from PubMed, Scopus and African Journals Online and references from relevant articles.

**Methods:**

Two reviewers independently analysed the studies using Arksey and O’Malley’s framework and Preferred Reporting Items for Systematic Reviews extension - Scoping Review guidelines. Using the most common solutions proposed for expanding KT in RLCs in the reviewed articles, a questionnaire was drafted to gain consensus among NPNs on solutions most suitable for the Nigerian setting. The NPNs used performance indicators, including efficiency, equity, quality of care, effects on catastrophic health expenditure and sustainability. To reach consensus, a solution had to satisfy all performance indicators and achieve at least 75% agreement among NPNs.

**Results:**

Of the 41 065 records identified, 39 articles were included for syntheses. Key barriers included inadequate financing and organ availability. The respondents comprised fourteen NPNs from 13 Nigerian clinical sites. Agreed solutions include the formation of a Nigeria Transplant Community to oversee PKT expansion, public KT centres (one per each of the six geopolitical zones), generic immunosuppressants and local dialysate production to cut costs.

**Conclusions:**

This study outlines a scalable pathway for PKT in Nigeria using a methodology adaptable to other RLCs. It emphasises government-backed financing and policy frameworks.

**WHAT IS ALREADY KNOWN ON THIS TOPIC**

- Paediatric kidney transplantation (PKT) rates in high-income countries range from 30 to 60 per million children, while in low- and middle-income countries (LMICs), rates fall below 1–5 per million, which contributes to preventable child deaths.

**WHAT THIS STUDY ADDS**

- This scoping review identifies solutions for improving access to kidney transplantation and PKT in LMICs and proposes a public-funded PKT model.

**HOW THIS STUDY MIGHT RESEARCH, PRACTICE OR POLICY**

- Through professional insights, the study emphasises the potential for establishing effective PKT in LMICs and highlights the need for equity. The proposed model is adaptable in LMICs, and success hinges on political will, multisectoral collaboration and addressing gaps in funding, healthcare availability, workforce and service quality for PKT.

## Introduction

 Kidney transplantation (KT) is the preferred kidney replacement therapy for children diagnosed with chronic kidney disease stage 5 (CKD-5).^1^ Paediatric KT (PKT) improves the quality of life and outcomes compared with dialysis; it supports children’s growth and development, is cost-effective for healthcare and ensures optimal long-term survival.^1^ The science of KT is improving rapidly in high-income countries (HICs) with precision medicine, genetic, diagnostic and pharmacodynamic accuracy, artificial intelligence and, more recently, xenotransplantation.^1 2^ The improved outcomes of KT in children globally represent advances in dialysis, surgical techniques, immunosuppressive therapy, treatment of allograft rejections, effective control of infections, prophylaxis for graft thrombosis and allograft size not encumbered by the donor’s size and age.^3 4^

The International Society of Nephrology’s Global Kidney Health Atlas of 2023 recently highlights significant disparities in access to PKT globally.^5^ Among 167 countries surveyed, KT services were available in 70% of all countries, including 86% of HICs, but only 21% of low-income countries (LICs). In 80% of countries, access to KT was greater in adults than in children.^5^ Pre-emptive KT remained exclusive to high- and upper-middle-income countries and living donor KT (LDKT) was the only available modality for KT in LICs. In HICs in North America, Western Europe and Australia, PKT rates reach 30–60 per million children population (pmcp), with 5-year graft survival rates exceeding 90% due to advanced care and reliance on deceased donors.^5^ Conversely, in low- and middle-income countries (LMICs) in most of Africa, South Asia and Latin America, PKT rates are below 1–5 pmcp, with over 90% relying on living donors.^5^ Tragically, children in LMICs are 10–20 times more likely to die on dialysis while waiting for transplants.^5 6^

Disparities in access to PKT are related to intracountry and intercountry differences in healthcare resources, workforce capacity, strategy development, regulatory oversight, economic status, race, ethnicity and geography.^7^ The COVID-19 pandemic has also further worsened these disparities.^8^ A country’s gross national income (GNI) per capita also impacts KT rates through proximate effects on its healthcare system, surveillance, service delivery, infrastructure and retention of the professional workforce required for KT programmes.^9^

The estimated incidence of CKD in Nigerian children ranges from 1.6 pmcp to 11 pmcp, and this is mostly secondary to nephrotic syndrome, glomerulonephritis and posterior urethral valves.^9^,^14^ Limited medical knowledge, screening efforts and restricted access to kidney care contribute as factors leading to a more rapid progression of CKD to kidney failure in Nigeria.^15^

In Nigeria, the minimum wage of workers is 70 000 Nigerian Naira per month^16^ (approximately US$44 or US$527 annually). Therefore, it is challenging for most Nigerians to afford the out-of-pocket costs of haemodialysis (HD) (US$23 096 USD annually) and LDKT (US$37 271), even if it is available.^17^ Nigeria’s National Health Insurance Scheme does not cover HD or KT for patients with CKD-5; <5% of the Nigerian population has access to health insurance, while 70% still finance their healthcare independently.^18 19^ A pooled analysis of five HD centres in Nigeria found that only 15% of CKD-5 patients can afford HD for more than 30 days.^20^ Thus, a diagnosis of CKD-5 is fatal for most Nigerian children.^11^

St. Nicholas Hospital in Lagos achieved Nigeria’s first successful KT in adult patients over 20 years ago in 2000.^21^ The first PKT occurred 9 years later, in 2009, at the same hospital, but it has been a sporadic service ever since.^22^ An estimated 7.5 pmcp develop CKD-5 annually in Nigeria,^11 23^ thus with 44% of Nigeria’s 200 million people being under 14 years of age, an estimated 400–500 children develop CKD-5 annually. Sadly, fewer than 0.2% of these children have access to PKT.^21^ Over the 33 years from 1986 to 2019, only 22 children received PKT, mostly in private hospitals or via transplant tourism to India.^21^ This is an average of 0.67 transplants/year. Major challenges include a lack of organ donors, no capacity for deceased donation KT (DDKT) and high treatment costs.^21^

Expanding PKT in Nigeria can leverage strengths built over the past 50 years. These include PKT experience in private hospitals^24^; the National Health Act (2014), which permits living and deceased donation^25^; the Nigerian Paediatric Renal Registry (NPRR, established 2019).^21^ Further strengths are professional support from the Paediatric Nephrology Association of Nigeria (PNAN),^21^ the Transplant Association of Nigeria^26^ and the Nigerian Association of Nephrologists (NAN).^27^ Collaborations with international organisations are also in place, including the International Society of Nephrology (ISN), the International Paediatric Nephrology Association (IPNA), the International Society of Peritoneal Dialysis (ISPD) and the International Paediatric Transplant Association (IPTA).^21^

In addition, Nigeria benefits from the existing progress and readiness to manage viral infections.^28^

However, establishing universal access and sustainable systems to support PKT requires an effective model to address the challenges that hinder its expansion in Nigeria.^1121 23^,^26 29^

This study aimed to conduct a scoping review to identify the challenges and solutions to improving access to PKT in resource-limited countries (RLCs) and derive consensus recommendations for Nigeria from a Nigerian paediatric nephrologists (NPNs) cohort.

## Methods

### Protocol, design, purpose and scope

This study used a scoping review to map evidence and derive consensus recommendations. Scoping reviews summarise available research, identify trends and consider implications for practice and policy.^30 31^ Accordingly, we synthesised studies on challenges and solutions for establishing and expanding KT/PKT in RLCs. Solutions that were frequently proposed and/or linked to sustained effectiveness and success in KT practices or improved access informed a structured questionnaire for adoption by NPNs.

### Definitions

**Figure 1:**
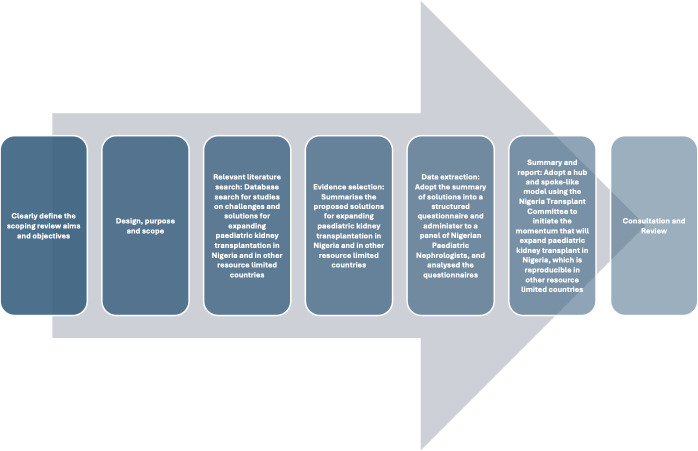
The study protocol of a scoping review to derive recommendations for establishing paediatric kidney transplantation in Nigeria.

Per World Bank definitions, RLCs comprise LICs (GNI per capita ≤ US$1135) and LMICs (GNI per capita of between US$1136 and US$4465).^32^ The protocol followed the Preferred Reporting Items for Systematic Reviews extension for Scoping Reviews (PRISMA-ScR) guidelines ^33 34^ and a seven-step process aligned to Arksey and O’Malley: (1) define aims/objectives; (2) design/purpose/scope; (3) identify literature; (4) select evidence; (5) extract/chart data; (6) collate, summarise, report; (7) consult/review (figure 1).^30^

### Identify literature

We searched PubMed, SCOPUS and African Journals Online for KT/PKT studies in Nigeria and other RLCs from January 2003 to December 2023. Because PKT in Nigeria and many RLCs is nascent and typically follows adult KT, ^5^ we included both adult KT and PKT literature. Reference lists of included articles were snowballed to identify additional records. ^35^ Search strings combined terms for challenges/solutions, KT/PKT, child/adult populations, RLC/LMIC/LIC geographies, financing and service delivery, using Boolean operators and database-specific syntax (online supplemental table 1).

### Evidence selection and data extraction

Inclusion: English-language articles comprising primary research, medical education/communication pieces, narrative reviews and expert opinions from nephrologists or health-policy stakeholders. Exclusion: unpublished private communications. Two independent reviewers screened titles/abstracts, then full texts; no conflicts required adjudication. Data were charted using PICO; *P*opulation: challenges affecting adult KT or PKT in RLCs; *I*ntervention: proposed solutions for KT/PKT and *C*omparison**/***O*utcome: evidence of success or failure (effectiveness, access, sustainability). Extracted fields included author/year, setting, design, barriers/enablers, interventions (eg, legislation, financing, workforce, infrastructure) and key outcomes. Findings were summarised by PICO to identify recurrent, feasible solutions and to note similarities/discrepancies across settings. Quantitative results were described using simple descriptive statistics; qualitative evidence underwent content analysis.

### Questionnaire development, administration and response analysis

The proposed solutions for the challenges of KT/PKT were summarised and prioritised, and a structured questionnaire was administered to determine consensus among NPNs to expand PKT in Nigeria. These NPNs are experts in addressing the challenges of providing care in a low-resource setting. Each proposed solution was assessed using performance indicators including efficiency,^36^ equity,^37^ quality of care,^38^ effects on catastrophic health expenditure^39^ and sustainability.^40^ In brief, efficiency in healthcare measures the relationship between resource inputs and health outcomes to achieve health system goals and obtain the best value for money.^36^ Equity refers to the absence of unfair, avoidable or remediable differences among groups of people based on various dimensions of inequality, including sex, gender, ethnicity, disability or sexual orientation.^37^ A good quality of care is characterised by being timely, effective, equitable, safe and people-centred. It is evidence-based, avoiding harm to those who receive it and responsive to individual preferences, needs and values.^38^ Catastrophic health expenditure describes a situation where health spending surpasses 40% of a household’s non-subsistence income, which can result in poorer treatment outcomes and reduced use of health services.^39^ The WHO defines a sustainable healthcare system as improving health while minimising negative environmental impacts and leveraging opportunities for future generations’ benefit.^40^ A solution must satisfy all performance indicators and attain a 75% agreement among the interviewees for a consensus to be reached. The interviewees/NPN’s contacts were obtained from the personal details of the NPNs available on the National Post-Graduate Medical College of Nigeria, the Faculty of Paediatrics and the WhatsApp groups of the H3 Africa Kidney Disease Research Network. Only NPNs currently practising in Nigeria and with ≥3 years of practice in nephrology were contacted to answer the structured questionnaire sent to them via the email addresses. The data were analysed through simple descriptive statistics, using mean and SD for continuous data and counts and percentages for categorical data. The consensus was determined according to a predefined threshold.

### Risk of bias assessment

Because this scoping review aspires to map all available evidence comprehensively, the authors refrained from conducting risk of bias assessments of included studies because of contextual differences in their healthcare system settings.^31 41^

## Results

### Summary, report and PRISMA flow

**Figure 2:**
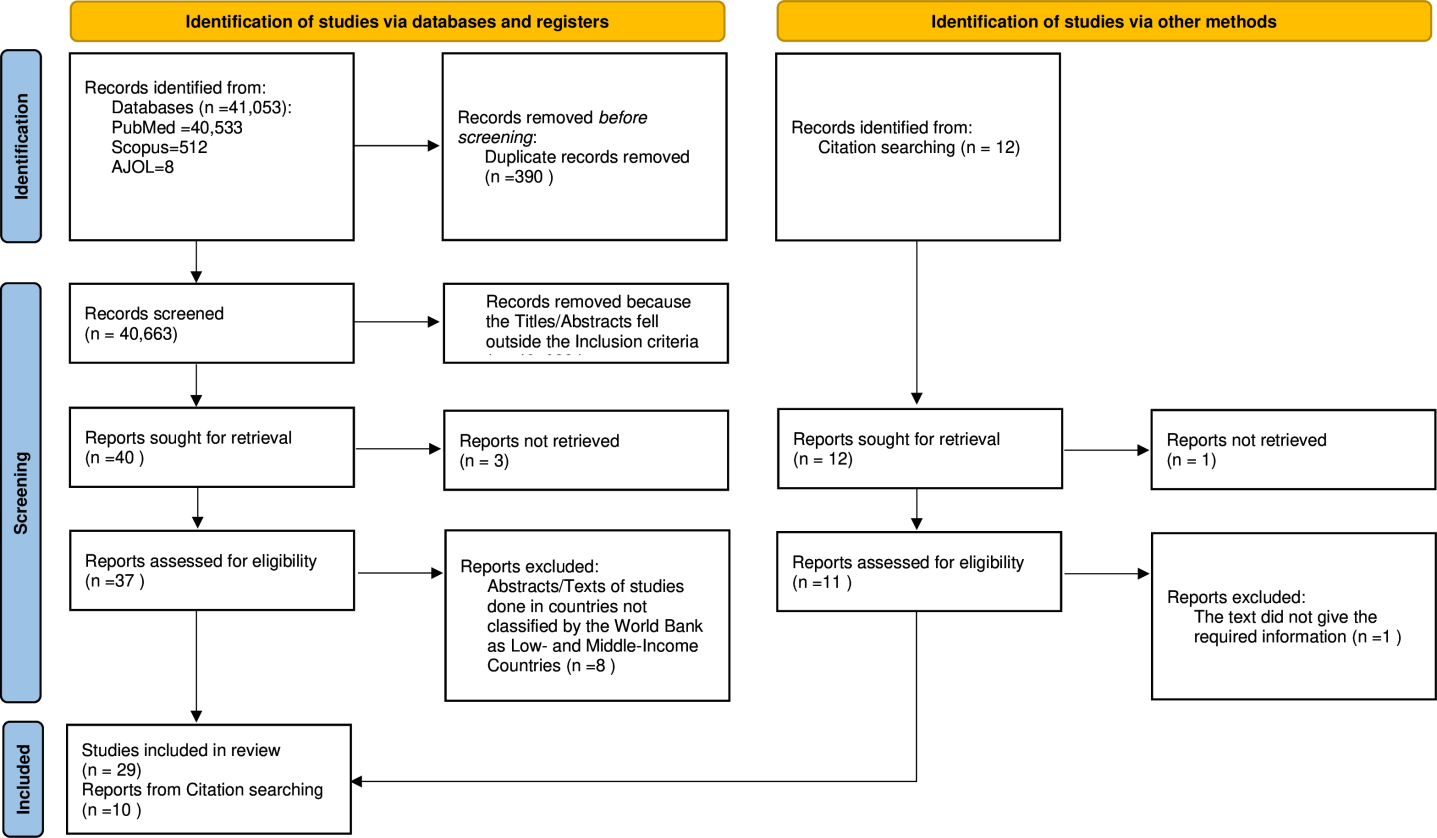
Preferred Reporting Items for Systematic Reviews flow of the included studies.

Figure 2 shows the PRISMA flow of the scoping review and the included. Of the 41 065 studies identified, 39 papers met the inclusion criteria and were reviewed. This included 22 (56.4%) studies from RLCs^2122 24^,^26 29 42^ and 17 (43.6%) publications of experts’ opinions.^58^,^73^ Specifically, six (15.4%) papers published focused on Nigeria,^2122 24^,^26 29^ five (12.8%) were from India,^47 50 52 55 57^ two (5.1%) from Pakistan^42 46^ and one each from Iran,^43^ Nepal,^44^ Morocco,^45^ Ivory Coast,^48^ Yemen,^49^ Tunisia,^51^ Rwanda,^53^ Egypt^54^ and Syria.^56^ A comprehensive evidence summary of the literature review, including the authors, year of publication of the article, settings, challenges of KT/PKT and solutions, is provided in online supplemental table 2 (published studies) and online supplemental table 3 (published expert opinions).

### Challenges in accessing and expanding PKT in RLCs

The capacity of RLCs to provide KT/PKT reported shared challenges, including inadequate government-backed public funding of KT/PKT,^2122 25 26 29 42 44^,^46 48^ inadequate infrastructural and transplant workforce support,^2122 24^,^26 29 42 44 45 47^ shortages of living and deceased organs,^2224 29 43 45^,^56 58^ inadequate legislative and regulatory framework for living and deceased KT^2122 24^,^26 47 48 50 52 53 58^ and uneven distribution of KT facilities and renal care services.^22 26 46 47 52 53 60 65 68 73^ The details of the barriers and the proposed solutions to expanding KT/PKT from the reviewed articles were as summarised in online supplemental table 4. Apart from South Africa, Morocco, Algeria and Tunisia, DDKT was unavailable in Nigeria and most other African countries.^68^ The data also showed that Nigeria, Ghana, Kenya, Sudan, Morocco, Tunisia and Algeria have some KT capabilities but low incidence rates.^71^ Sudan has a successful KT programme owing to a well-developed health system and strong government and health-insurance support for KT financing.^71^ In contrast, Nigeria^21 22 24 26 29 51^ and Ivory Coast^48^ depend on private OOP payments for KT, creating significant barriers to KT for many patients.

Rizvi *et al*^46 74 75^ reported that Pakistan’s integrated renal care programme, based on a community-government partnership, has achieved long-term success. Experts like Saeed,^69^ Iyengar and McCulloch^70^ and Mudiayi *et al*^71^ advocated adopting this model in other RLCs. Ackoundou-N’Guessan *et al* and Amira *et al* have also advocated for the adoption of the Pakistan model in Ivory Coast and Nigeria, respectively.^25 48^ The Pakistani programme has thrived for over 40 years, with the government covering infrastructure and half of the operating costs, while the community, including wealthy individuals and corporations, provides the rest.^46 74 75^ In contrast, Iran’s regulated system for monetary compensation for living unrelated donations, which effectively eliminated waiting lists by addressing ethical concerns, has not been adopted in other RLCs.^43^

### Questionnaire analysis

**Figure 3:**
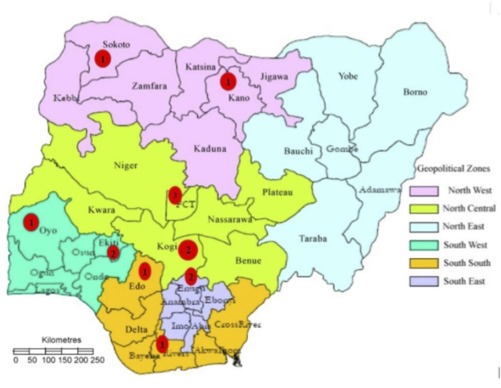
The spatial distribution of the 14 respondents (red circles) across the geopolitical regions of Nigeria.

The request to contribute to this project was sent to seventeen NPNs, of which 13 (76.5%) responded. The four NPNs that could not return the questionnaire survey included one each from the Southwest, Southeast, Northcentral and Northwest geopolitical regions of Nigeria, which were also incidentally well represented by the NPNs that returned the questionnaires. These respondents, consisting of 14 NPNs, were from five of the six geopolitical regions of Nigeria, as depicted in figure 3. Unfortunately, there was a paucity of respondents from the Northeast geopolitical region of Nigeria, which was linked to the maldistribution of the healthcare workforce.^76^ 13 NPNs were working in government-owned public tertiary hospitals, and one was working in a private hospital (Zenith Medical and Kidney Centre, Abuja) that was most actively engaged in PKT in Nigeria. Each panellist’s experience practising paediatric nephrology ranged from 4 years to 32 years (mean 14.0±6.3 years).

## Recommendations for expanding PKT in Nigeria

A summary analysis of the consensus achieved is shown in online supplemental table 5. The NPNs made seven overarching recommendations, as summarised in box 1. The details included an agreement (78.5%) to amend the existing Nigerian regulatory legislation^27^ on living and deceased donation, compulsory follow-up care for living donors, standardisation of healthcare facilities that can provide transplantation, the establishment of Organ Donor Foundation to increase organ pool via expanded criteria and transplant of blood-group incompatible pairs, optimising financing for government-public funded PKT, freeing up fiscal space to increase health financing, structural removal of inequitable subsidies that are limiting the government’s available financial resources, use of generic immunosuppressants and agreement (78.5%) against commodification of human organ/tissue and financial inducement for a living donor. The respondents also agreed (92.9%) that the living donor must be counselled on the risks of donation and that there must be compulsory care for living donors in case of untoward consequences (85.7%). They agreed (85.7%) to a paired exchange programme, to recognise deceased donation as a noble act (92.9%) and for PKT to be prioritised over adult KT in organ allocation for deceased donation (85.7%). Regarding financing comprehensive care for patients with CKD/CKD-5, including KT, the respondents agreed (85.7%) that through lobbying, the proposed Nigeria Transplant Community (NTC) should advocate for the budgetary allocation for the health of not less than 15% of the country’s Consolidated Revenue Fund, meaning the Basic Health Care Provision Fund (BHCPF) would include care for CKD/CKD-5 (85.7%). They also agreed (85.7%) to form a parallel stand-alone National Renal Care Fund (NRCF) to care for CKD/CKD-5, including KT. Moreover, to increase the funds available for renal care, the respondents agreed (85.7%) to impose taxes on companies and levies on commercial services (85.7%). Additionally, it was agreed (85.7%) to encourage public-private care funding for CKD/CKD-5/KT through community-based non-governmental philanthropic schemes and crowdfunding. Regarding cost-saving measures, the respondents agreed (85.7%) to the use of generic calcineurin inhibitors (CNIs) (78.5%), combining metabolic inhibitors with CNIs (85.7%), lobbying to enlist immunosuppressants as essential medicines (92.9%), in-country production of dialysis fluid (92.9%) and the use of basic dialyser machines (78.5%). However, the respondents disagreed with the use of azathioprine instead of mycophenolate (71.4%), the reuse of dialysers (64.3%) and pre-emptive arterio-venous fistula construction (64.3%) as cost-saving measures.

Box 1 **Overarching recommendations for starting and expanding paediatric kidney transplantation programmes in resource-limited countries**

- Generate momentum to form a Transplant Community.
- Optimise government funding, financing and reimbursement for paediatric kidney transplantation.
- Start or keep the Paediatric Kidney Registry for data monitoring and surveillance for effective policy and governance.
- Optimise ethical and regulatory legislation for living donation including organ pool.
- Optimise legal, ethical and regulatory frameworks for deceased donation.
- Build paediatric kidney transplantation workforce.
- Build health infrastructure at the proposed paediatric kidney transplantation centres.

While the respondents agreed (92.9%) on the need to build a specific kidney transplant workforce via international collaboration with the African Association of Nephrology (AFRAN), the ISN, the Transplant Society (TTS), the IPNA and the IPTA, they disagreed (64.3%) on task shifting among allied health professionals. Finally, the respondents (92.9%) agreed that one publicly owned PKT centre be established in each of the country’s six geopolitical regions. They suggested gradually building expertise by starting with LDKT before transitioning to DDKT.

## Discussion

Children with CKD-5 continue to die unnecessarily in LMICs, and the need for increasing access to PKT cannot be overstated.

The ISN framework for integrated kidney care prioritises KT above other methods of kidney replacement therapy.^77^ Despite its relatively high cost, KT is a life-saving procedure well-established in some RLCs.^78^

However, strong commitment from the national government, medical practitioners and health authorities is essential in achieving a sustainable KT programme in these complex settings.^78^ The ideal PKT programme should be publicly funded to provide equitable access regardless of socioeconomic status, religion, creed, gender or age.^63^ This work agrees with the Kidney Disease: Improving Global Outcomes Clinical Practice Guideline on the Evaluation and Care of Living Kidney Donors.^79^ The guideline identifies that an ideal LDKT programme should have a multidisciplinary team, follow standardised operations, identify patients for pre-emptive KT, manage resources effectively, prioritise suitable living donors and ensure informed consent.

A government-funded KT programme will fund or reimburse all aspects of transplant care, including pretransplant clinical evaluation, transplant surgery, post-transplant hospital admissions, rehabilitation, induction, long-term immunosuppressive therapy and follow-up.^79 80^

For countries with established LDKT programmes, a reasonable goal is to have >80% of children with CKD-5 receive KT when medically feasible.^79^

Akin to the Nigerian nephrologists’ recommendations on expanding KT in Nigeria, made a decade ago,^80^ the NPNs in this study made the overarching recommendations depicted in box 1. It is important to note that these overarching recommendations are adaptable pathways that can help expand and increase accessibility to PKT in other RLCs with similar challenges to Nigeria.^81^ Thus, other RLCs with some capacities for KT but which are plagued with low incidence rates will be able to expand PKT service delivery with these recommendations.

**Figure 4:**
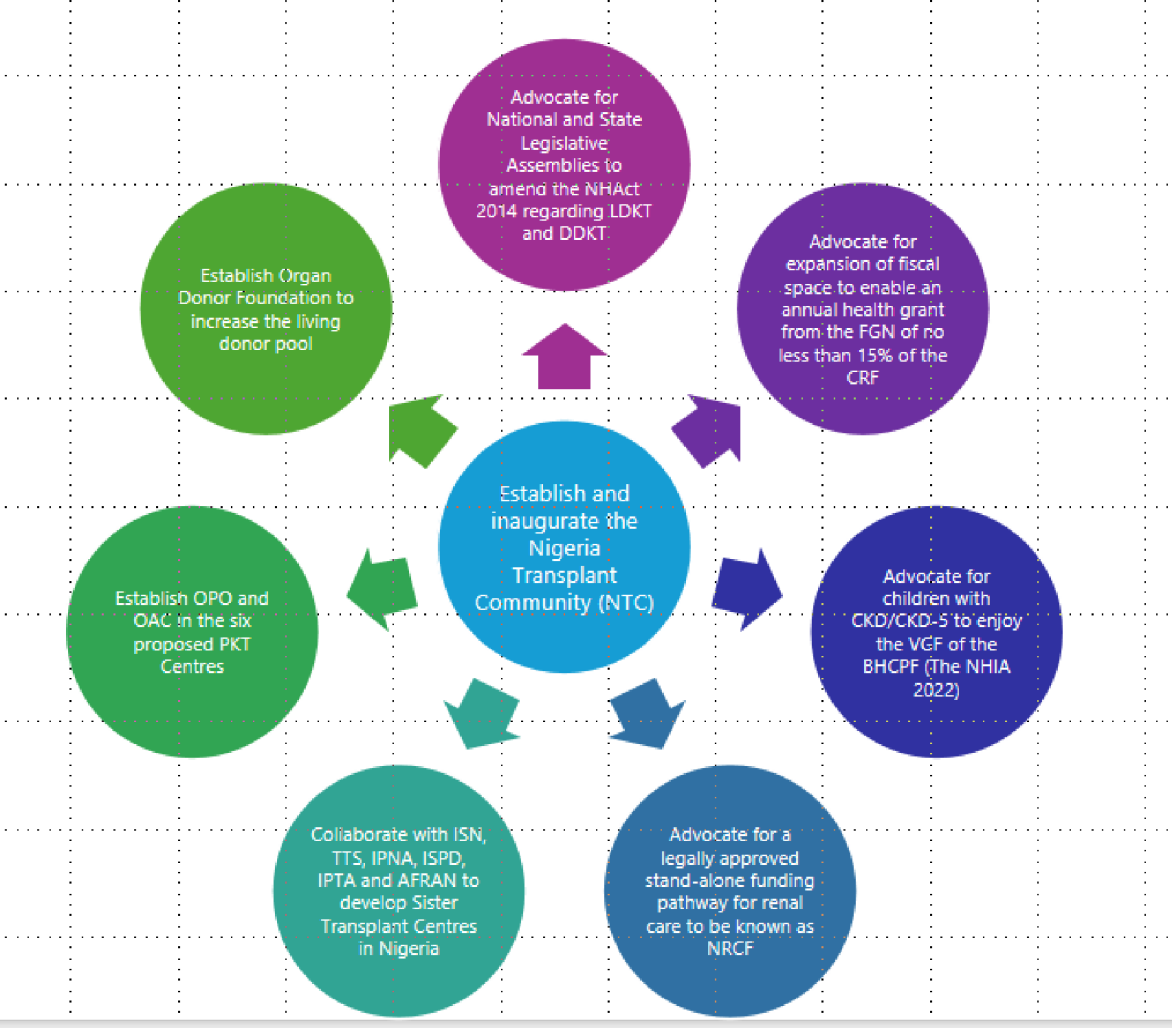
A hub and spoke-like model of implementing expansion of paediatric kidney. AFRAN, African Association of Nephrology; BHCPF, basic Basic health careHealth Care provision fun Provision Fund; CKD, chronic kidney disease; CKD-5, CKD stage 5; CRF, Consolidated Revenue Fund; DDKT, deceased donation KT; FGN, Federal Government of Nigeria; IPNA, International Paediatric Nephrology Association; ISPD, International Society of Peritoneal Dialysis; ISN, International Society of Nephrology; KT, kidney transplantation; LDKT, living donor KT; NHAct, National Health Act 2014; NHIA, National Health Insurance Authority; NRCF, National Renal Care Fund; OAC, organ allocation committee; OPO, organ procurement organisation; PKT, paediatric KT; TTS, The Transplant Society; VGF, Vulnerable Group Fund

To successfully deliver these recommendations, the NPNs will prioritise the formation of the NTC. The NTC shall include members of the PNAN, the TAN and the NAN, paediatric-trained nurses, dietitians, psychiatrists, pharmacists, histopathologists, radiologists, laboratorians, anaesthetists, intensivists, transplant surgeons, social workers and others—non-governmental organisations, media and leaders from religious bodies (Christian Association of Nigeria, Nigerian Supreme Councils for Islamic Affairs, Traditional Religion Association). The NTC shall be the coordinator and form the National Coordinating Committee for the PKT programme. It is anticipated that in a hub and spoke-like model, the NTC shall be the hub that will coordinate other KT activities (figure 4). Among other responsibilities, the NTC shall lobby the Honourable Minister of Health and other significant stakeholders to amend the National Health Act 2014 regarding living and deceased organ donations.^27^ The significant stakeholders comprise the National Council on Health, Nigeria’s highest health policy-making body.^27^ Other significant stakeholders include the National/States Legislative Assemblies, the Federal Ministry of Health, the National Council on Health Technical Committee and the National Tertiary Health Institutions Standard Committee.

The NTC will also collaborate with international organisations such as the ISN, TTS, IPNA, ISPD, IPTA and AFRAN to develop ‘Sister Transplant Centres’ in the proposed PKT centres in Nigeria. Through international collaboration, professional training fellowships and clinical guidelines will be shared. The NTC will help establish an organ procurement organisation and an organ allocation committee in the proposed PKT centres.

The greatest obstacle to PKT identified in Nigeria^12 21 22 25 26 29^ and other RLCs^52 55 58 62 63 65^ is inadequate government funding. For families, the OOP health expenditure associated with kidney replacement therapy often causes household poverty.^21 22 24 52 55 58 62 63 65^ There are various ways the NTC will lobby to increase government and public financing of PKT. These strategies include expanding the BHCPF^82^ to fund PKT services at the six proposed PKT centres through the National Health Insurance Authority gateway.^83^ The NTC will advocate for children and adults with CKD/CKD-5 to be classified as vulnerable, making them eligible for the Vulnerable Group Fund under the BHCPF.^82^ Alternatively, the NTC will lobby the significant stakeholders to enact a law that will establish a stand-alone NRCF. The idea of having NRCF is premised on providing funds for the National Renal Care Policy, which the NAN has proposed.^84^ The NTC must advocate for the expansion of fiscal space. This can be done by campaigning for an annual financial allocation from the Federal Government of Nigeria of not less than 15% of the Consolidated Revenue Fund to fund health. Significant stakeholders can be lobbied to increase the financial resources needed to fund health in Nigeria. Companies can be subjected to special PKT taxation via their profits and capital gains as part of their corporate social responsibilities. Commercial services such as mobile phone use, financial transactions and air travel can be subjected to special PKT levies. Through public campaigns, the NTC shall encourage public funding via community-based non-governmental philanthropic fund schemes and crowdfunding. On the clinical front, the NTC shall support using generic CNIs and ketoconazole to reduce medication costs. The NTC will push immunosuppressants to be classified as essential medicines and promote government-funded chronic HD services in Nigeria. These measures align with the WHO’s proposal that CKD management should be part of a country’s universal healthcare.^85^ However, if it is not feasible for the national government to solely fund KT, government-public partnerships have also been reported to enhance access to KT service delivery effectively. 2546 48 69,71 74 75^.^

Before the NPRR was launched in 2019, the absence of comprehensive health information systems in Nigeria significantly hindered the capturing of essential data on PKT.^21^ The renal registry documents transplant activity, waiting lists, immediate and long-term outcomes of PKT and donor morbidity and mortality in the proposed system. A well-functioning national renal registry discourages organ commercialism and trafficking via close data monitoring and surveillance for effective policy and governance.^21 22 52 55 58 62 63 65^

There is a shortage of living donors as people often hesitate to donate.^12 21 22 25 26 29^ Education and awareness campaigns will aim to increase the number of kidney donors and uphold stringent ethical standards while respecting cultural beliefs.^21 22 52 55 58 62 63 65^ To expand organ donation activities, the NTC will use all the existing networks. These networks include the PNAN and TAN (established in 2008), the Kidney Transplant Advocacy Group and the Rekiff Kidney Support Group (founded in 2016).^21^ The Nigerian National Health Act 2014 legal, regulatory and ethical framework protects the integrity of living donation.^27^ This Act stipulates that living donors must be at least 18 years of age, prohibits organ or tissue sales and expects organ donations to occur in licensed hospitals under qualified supervision.^27^ The NTC will advocate for amendments to the Act that include mandatory counselling, follow-up care and support for complications that may follow organ donations. The NTC also aims to expand the donor pool through an Organ Donor Foundation, allowing incompatible transplants, expanded donor criteria and paired exchanges.

The definition of brain death is essential for deceased donation, and a patient determined to be brain dead is legally and clinically dead.^86^ The transition of paediatric LDKT to DDKT must also happen within a regulatory legislative framework. Inadequate legislation on brain death, a lack of dialysis access, inadequate tissue typing and crossmatching facilities, and a shortage of emergency organ harvesting surgeries and coordination are among the factors limiting DDKT.^21 22 24 52 58 62 70^ Some favourable regulatory legislative Acts already exist supporting DDKT in Nigeria.^27^ In this study, the NPNs agreed on this existing legislation, including provisions that competent individuals can donate their body or tissues after death through written statements or wills, donors can specify the recipient(s) of their donations on demise, and organs from deceased donors can be transplanted into living individuals. However, the NTC will lobby the significant stakeholders to amend the existing Act to allow for recognition of donors during funeral services and a posthumous national award, advocating for priority allocation of organs from deceased donors to paediatric patients, establishing organ procurement organisations in the proposed PKT centres to manage organ pool, and the formation of organ allocation committees to manage and allocate organs. The NTC shall also engage in public enlightenment campaigns to address cultural and religious biases against deceased donations. However, to ensure a seamless DDKT process, Nigeria needs to develop infrastructure that supports deceased donation, including having waiting lists, transplant coordinators, on-call transplantation teams and relevant emergency intensive care resources.^21 87^

In RLCs, including Nigeria, the overall health infrastructure is weak, so PKT is often managed by adult nephrologists.^21 70^ Paediatric nephrologists are lacking and transplant surgeons from overseas are relied on in some centres.^21 24 25^ The distribution of dialysis centres is also uneven, adding further barriers.^21 22 24 65^ For this proposal to be successful, a more trained transplant workforce is needed. PKT requires transplant surgeons, nephrologists, anaesthesiologists, intensivists, skilled nurses, pharmacists, histopathologists and scientists to run an HLA laboratory.^26 63 70^ The NTC will advocate for Nigeria’s Post-graduate Medical College and the West African College of Physicians (Paediatrics) to make PKT science mandatory in their post-doctoral training curriculum. The NTC will also collaborate with IPNA, IPTA, TTS and ISN to continue the fellowship training programmes explicitly targeted at PKT. The NTC will help to develop training modules and protocols for PKT. To enhance health infrastructure at PKT centres, the NTC will prioritise LDKT first. Once established, the focus will shift to DDKT services.

Finally, the NPNs in this study agreed that one government-owned KT centre should be established in each of the country’s six geopolitical regions for equitable access. The NTC will ensure that the National Tertiary Health Institution Standard Committee provides standards for approving KT facilities and granting certification. Other RLCs with similar settings as Nigeria wishing to have an equitable PKT service can benefit from strategies with moderate success in Nigeria. At the same time, they learn from the country’s failure. These successes include leveraging KT experience already available for PKT in a few private hospitals as well as adult KT services operational in some government-owned hospitals, enacting national regulatory legislation that supports living and deceased KT, having a practical and functional paediatric renal registry and collaborating with international organisations such as ISN, IPNA and TTS for training local transplant workforce through Sister Transplant Centres Programme.^1121^,^28 65^ However, other RLCs can also learn from barriers that mitigate against PKT expansion in Nigeria. A significant obstacle remains poor budgetary allocation to health, with no specific allocation for CKD/CKD-5 patients. Healthcare spending per capita in 2025 is US$7.8; it remains critically low, equating to US$0.02 per day, accounting for only 5.15% of the total budget, significantly below Africa’s target of 15% (The Abuja Declaration).^88 89^

Healthcare workforce migration is another major challenge of PKT in Nigeria, with the country losing trained doctors overseas.^65^ Skilled migration can be minimised when countries increase investment in healthcare, provide better job satisfaction and standard of living, and increase income for the healthcare workforce.^65^

The study’s limitations include the fact that the consensus data were collected through questionnaires. However, they were created using the best available evidence, and this was the most realistic method to ensure maximum engagement from the diverse setting. The NPNs’ expertise, experience and knowledge limitations may have also influenced the study outcome, and their perceptions may not necessarily reflect the views of the broader population. This study does not guarantee that these solutions can be integrated into Nigeria’s context. Despite the limitations, this study is a landmark for accelerating life-saving kidney care for children in Nigeria. From this study, the strategy will be broken down into achievable goals with deliverable milestones and tasks shared among NTC members. A significant strength of this article is that it checked all 22 items on the PRISMA-ScR (online supplemental table 6).^34^

## Consultation and review

The authors ensure that consultation and review incorporate diverse perspectives, keep the review focused and communicate findings effectively.

## Conclusions

The burden of CKD-5 among children’s populations is increasing globally and in Nigeria. Despite the overwhelming challenges in establishing a successful kidney care system for children in RLCs, this study has outlined a pathway to establish and expand PKT services. This strategy will require concerted efforts driven by the NTC. This study hopes to be the first important step in making KT universally accessible to Nigerian children and, by doing so, save many young lives.

## Supplementary material

10.1136/bmjgh-2024-017023 online supplemental file 1

10.1136/bmjgh-2024-017023 online supplemental file 2

10.1136/bmjgh-2024-017023 online supplemental file 3

10.1136/bmjgh-2024-017023 online supplemental file 4

10.1136/bmjgh-2024-017023 online supplemental file 5

10.1136/bmjgh-2024-017023 online supplemental file 6

## Data Availability

All data relevant to the study are included in the article or uploaded as supplementary information.

## References

[1] Pickles CW, Brown C, Marks SD (2023). Long term outcomes following kidney transplantation in children who weighed less than 15 kg - report from the UK Transplant Registry. Pediatr Nephrol.

[2] Eissa N, M. Badrkhan S, A. Mohamed M (2022). Xenotransplantation: past, present, and future directions. Highlights in BioScience.

[3] Ghelichi-Ghojogh M, Mohammadizadeh F, Jafari F (2022). The global survival rate of graft and patient in kidney transplantation of children: a systematic review and meta-analysis. BMC Pediatr.

[4] Fernandez HE, Foster BJ (2022). Long-Term Care of the Pediatric Kidney Transplant Recipient. Clin J Am Soc Nephrol.

[5] Viecelli AK, Gately R, Barday Z (2024). Worldwide organization and structures for kidney transplantation services. Nephrol Dial Transplant.

[6] Stel V, Ortiz A (2023). ERA registry report. https://www.era-online.org/wp-content/uploads/2024/04/ERA-Registry-Annual-Report-2023.pdf.

[7] Francis A, Harhay MN, Ong ACM (2024). Chronic kidney disease and the global public health agenda: an international consensus. Nat Rev Nephrol.

[8] Charnaya O, Chiang TP-Y, Wang R (2021). Effects of COVID-19 pandemic on pediatric kidney transplant in the United States. Pediatr Nephrol.

[9] Mudiayi D, Shojai S, Okpechi I (2022). Global Estimates of Capacity for Kidney Transplantation in World Countries and Regions. Transplantation.

[10] Anigilaje EA, Kanu AO (2017). Chronic Kidney Disease in Children at the University of Abuja Teaching Hospital Abuja, Nigeria. Trop J Nephrol.

[11] Asinobi AO, Ademola AD, Ogunkunle OO (2014). Paediatric end-stage renal disease in a tertiary hospital in South West Nigeria. BMC Nephrol.

[12] Odetunde OI, Okafor HU, Uwaezuoke SN (2014). Chronic kidney disease in children as seen in a tertiary hospital in Enugu, South-East, Nigeria. Niger J Clin Pract.

[13] Anochie I, Eke F (2003). Chronic renal failure in children: a report from Port Harcourt, Nigeria (1985-2000). Pediatr Nephrol.

[14] Olowu WA, Adefehinti O, Aladekomo TA (2013). Epidemiology and clinicopathologic outcome of pediatric chronic kidney disease in Nigeria, a single cenetr study. Arab J Nephrol Transplant.

[15] Arogundade FA, Esezobor CI, Okafor HU, Moura-Neto JA, Divino-Filho JC, Ronco C (2021). Nephrology worldwide.

[16] (2024). The national minimum wage (amendment) act. https://www.mjnuma.com/newsletter/pdfs/43.pdf#:~:text=The%20most%20significant%20change%20in%20the%20National%20Minimum,repealed%202019%20Act%20to%20%E2%82%A670%2C000%20%28Seventy%20Thousand%20Naira%29.

[17] Lang JJ, Lombardi CV, James IA (2022). A Payer’s Perspective: A Comparison and Simulation of the Costs of Hemodialysis Versus Living Donor Kidney Transplant for Patients With End-Stage Renal Disease in Nigeria. Transpl Int.

[18] Agada-Amade YA, Ogbuabor DC, Obikeze E (2024). Cost-benefit analysis of haemodialysis in patients with end-stage kidney disease in Abuja, Nigeria. Health Econ Rev.

[19] Eze OI, Iseolorunkanmi A, Adeloye D (2024). The National Health Insurance Scheme (NHIS) in Nigeria: current issues and implementation challenges. Journal of Global Health Economics and Policy.

[20] Liman HM, Sakajiki AM, Makusidi MA (2021). Public-private partnership in hemodialysis in Nigeria: A comparative analysis of renal centers across three Northwestern states. Ann Afr Med.

[21] Eke FU, Ladapo TA, Okpere AN (2021). The current status of kidney transplantation in Nigerian children: still awaiting light at the end of the tunnel. Pediatr Nephrol.

[22] Ulasi II, Ijoma CK (2016). Organ Transplantation in Nigeria. Transplantation.

[23] Eke FU, Eke NN (1994). Renal disorders in children: a Nigerian study. Pediatr Nephrol.

[24] Arogundade FA (2013). Kidney transplantation in a low-resource setting: Nigeria experience. Kidney Int Suppl (2011).

[25] (2017). National health act 2014. https://www.publichealth.com.ng/wp-content/uploads/2017/10/The-Official-Gazette-of-the-National-Health-Act.pdf.

[26] Popoola AA, Olanrewaju TO, Bolaji BO (2018). Expanding renal transplantation organ donor pool in Nigeria. Saudi J Kidney Dis Transpl.

[27] Amira CO, Busari AA, Bello BT (2017). Challenges accessing kidney transplantation in Lagos,Nigeria. *Niger J Health Sci*.

[28] Abubakar I, Dalglish SL, Angell B (2022). The Lancet Nigeria Commission: investing in health and the future of the nation. Lancet.

[29] Okafor UH (2016). Kidney transplant in Nigeria: a single centre experience. Pan Afr Med J.

[30] Arksey H, O’Malley L (2005). Scoping studies: towards a methodological framework. Int J Soc Res Methodol.

[31] Tricco AC, Lillie E, Zarin W (2016). A scoping review on the conduct and reporting of scoping reviews. BMC Med Res Methodol.

[32] (2023). Low or lower middle-income countries (LMIC) world bank list.

[33] Peters MDJ, Marnie C, Tricco AC (2020). Updated methodological guidance for the conduct of scoping reviews. *JBI Evid Synth*.

[34] Tricco AC, Lillie E, Zarin W (2018). PRISMA Extension for Scoping Reviews (PRISMA-ScR): Checklist and Explanation. Ann Intern Med.

[35] Felizardo KR, Mendes E, Kalinowski M (2016). Using forward snowballing to update systematic reviews in software engineering. https://dl.acm.org/doi/proceedings/10.1145/2961111.

[36] Yip W, Hafez R (2015). Improving health system efficiency. http://apps.who.int/iris/bitstream/10665/185989/1/WHO_HIS_HGF_SR_15.1_eng.pdf?ua=1.

[37] (2023). Quality of care. https://hlh.who.int/quality-of-care.

[38] (2023). Health equity. https://www.who.int/health-topics/universal-health-coverage/health-equity.

[39] WHO (2005). Technical briefs for policy makers. designing health financing systems to reduce catastrophic health expenditure. https://www.who.int/publications/i/item/WHO-EIP-HSF-PB-05.02.

[40] (2017). Environmentally sustainable health systems: a strategic document (WHO.int). https://www.who.int/publications/i/item/WHO-EURO-2017-2241-41996-57723.

[41] Peters M, Godfrey C, McInerney P (2015). The Joanna Briggs institute reviewers manual 2015.

[42] Sakhuja V, Sud K (2003). End-stage renal disease in India and Pakistan: Burden of disease and management issues. Kidney Int.

[43] Ghods AJ, Savaj S (2006). Iranian model of paid and regulated living-unrelated kidney donation. Clin J Am Soc Nephrol.

[44] Chalise PR, Shah DS, Sharma UK (2010). Renal transplantation in Nepal: the first year’s experience. Saudi J Kidney Dis Transpl.

[45] Haddiya I, Radoui A, Benamar L (2012). Ten years of renal transplantation in a Moroccan hospital: results and constraints. Transplant Proc.

[46] Rizvi SAH, Sultan S, Zafar MN (2013). Pediatric kidney transplantation in the developing world: challenges and solutions. Am J Transplant.

[47] Kute VB, Vanikar AV, Shah PR (2014). Increasing access to kidney transplantation in countries with limited resources: the Indian experience with kidney paired donation. Nephrology (Carlton).

[48] Ackoundou-N’Guessan C, Hoang AD, Ben Abdallah T (2015). Living Kidney Donor Transplantation in a Resource-limited Country: The Ivory Coast Experience. Transplant Proc.

[49] El-Nono IH, Telha KA, Al-Alimy GM (2015). Challenges in renal transplantation in Yemen. Ann Transplant.

[50] Abraham G, Vijayan M, Gopalakrishnan N (2016). State of deceased donor transplantation in India: A model for developing countries around the world. World J Transplant.

[51] Abderrahim E, Zammouri A, Bacha MM Thirty Years of Experience at the First Tunisian Kidney Transplant Center. Exp Clin Transplant.

[52] Sethi SK, Sinha R, Rohatgi S (2017). Pediatric renal transplant practices in India. Pediatr Transplant.

[53] Chironda G, Ngendahayo F, Mudasumbwa G (2019). Renal replacement therapy (RRT) in Rwanda: benefits, challenges and recommendations. Rwanda Med J.

[54] Bakr MA, Shehab El-Dein AB, Refaie AF (2020). Renal Transplantation in Mansoura, Egypt. Transplantation.

[55] Kute V, Ramesh V, Shroff S (2020). Deceased-Donor Organ Transplantation in India: Current Status, Challenges, and Solutions. Exp Clin Transplant.

[56] Saeed B (2020). Organ Transplantation in Syria. Transplantation.

[57] Pais P, Blydt-Hansen TD, Michael Raj JA (2021). Low renal transplantation rates in children with end-stage kidney disease: A study of barriers in a low-resource setting. Pediatr Transplant.

[58] Persy VP, Remuzzi G, Perico N (2010). Prevention and transplantation in chronic kidney disease: what is achievable in emerging countries? Meeting report: Bamako meeting December 4-6, 2008. Nephron Clin Pract.

[59] Akoh JA (2011). Renal transplantation in developing countries. Saudi J Kidney Dis Transpl.

[60] Spearman CWN, McCulloch MI (2014). Challenges for paediatric transplantation in Africa. Pediatr Transplant.

[61] Muralidharan A, White S (2015). The need for kidney transplantation in low- and middle-income countries in 2012: an epidemiological perspective. Transplantation.

[62] Muller E (2016). Transplantation in Africa - an overview. Clin Nephrol.

[63] O’Connell PJ, Brown M, Chan TM (2020). The role of kidney transplantation as a component of integrated care for chronic kidney disease. Kidney Int Suppl (2011).

[64] Loua A, Feroleto M, Sougou A (2020). A review of policies and programmes for human organ and tissue donations and transplantations, WHO African Region. Bull World Health Organ.

[65] Esezobor CI, Alakaloko AE, Admani B (2021). Paediatric Nephrology in Africa. Curr Pediatr Rep.

[66] Elrggal ME, Gokcay Bek S, Shendi AM (2021). Disparities in Access to Kidney Transplantation in Developing Countries. Transplantation.

[67] Okpechi I, Niang A, Hafez M (2022). A roadmap for kidney care in Africa: An analysis of International Society of Nephrology–Global Kidney Health Atlas Africa data describing current gaps and opportunities. *ajn*.

[68] Bamgboye EL, Yadla M, Garcia-Garcia G (2022). Transplant: The Success of Renal Transplant Programs. Semin Nephrol.

[69] Saeed B (2022). Pediatric Kidney Transplantation in the Middle East: Challenges and Solutions. Exp Clin Transplant.

[70] Iyengar A, McCulloch MI (2022). Paediatric kidney transplantation in under-resourced regions-a panoramic view. Pediatr Nephrol.

[71] Mudiayi D, Shojai S, Okpechi I (2022). Global Estimates of Capacity for Kidney Transplantation in World Countries and Regions. Transplantation.

[72] Roberts LE, Kaur A, Jewitt-Harris J (2024). Kidney transplantation in low- and middle-income countries: the Transplant Links experience. Pediatr Nephrol.

[73] Vo HD, Mackie F, McCulloch M (2024). International pediatric transplant association (IPTA) guidance on developing and/or expanding pediatric solid organ transplantation programs in low- and middle-income countries. Pediatr Transplant.

[74] Rizvi SAH, Naqvi SAA, Zafar MN (2011). A renal transplantation model for developing countries. Am J Transplant.

[75] Zafar MN, Rizvi SAH (2023). Providing “Free” Access to Dialysis and Transplant to the Disfranchised. A Sustainable Model for Low and Low Middle Income Countries (LMICs). Transpl Int.

[76] Ekure E, Esezobor C, Balogun M (2013). Paediatrician workforce in Nigeria and impact on child health. Nig J Paed.

[77] Tonelli M, Nkunu V, Varghese C (2020). Framework for establishing integrated kidney care programs in low- and middle-income countries. Kidney Int Suppl (2011).

[78] Zafar MN, Wong G, Aziz T (2018). Living donor risk model for predicting kidney allograft and patient survival in an emerging economy. Nephrology (Carlton).

[79] Chadban SJ, Ahn C, Axelrod DA (2020). KDIGO Clinical Practice Guideline on the Evaluation and Management of Candidates for Kidney Transplantation. Transplantation.

[80] (2013). Report of the global alliance for transplantation workshop. https://sats.org.za/wp-content/uploads/2024/09/GAT-Report.pdf.

[81] Ogieuhi IJ, Aderinto N, Olatunji G (2024). Towards equitable renal care: Strategies for enhancing kidney transplantation in Africa. J Med Surg Public Health.

[82] Basic Healthcare Provision Fund (2020). Guideline for the administration, disbursement and monitoring of the basic health care provision fund (bhcpf). https://health.gov.ng/doc/BHCPF-2020-Guidelines.pdf.

[83] (2022). National health insurance authority act, 2022. https://nascpay.com/national-health-insurance-scheme-act-pdf.

[84] (2022). Kidney: nephrologist makes case for national renal care policy. https://independent.ng/kidney-nephrologist-makes-case-for-national-renal-care-policy/.

[85] World Health Organization (2017). Best buys’ and other recommended interventions for the prevention and control of noncommunicable diseases. https://iris.who.int/bitstream/handle/10665/259232/WHO-NMH-NVI-17.9-eng.pdf.

[86] Anwar A, Lee J-M (2019). Medical Management of Brain-Dead Organ Donors. *Acute Crit Care*.

[87] White SL, Hirth R, Mahíllo B (2014). The global diffusion of organ transplantation: trends, drivers and policy implications. *Bull World Health Organ*.

[88] Development Research and Projects Centre (dRPC) (2025). Understanding the nigeria 2025 proposed budget: the healthcare sector in focus. https://drpcngr.org/wp-content/uploads/2025/01/Understanding-the-Nigeria-2025-Proposed-Health-Budget-.pdf#:~:text=Healthcare%20spending%20per%20capita%20increased%20from%20%E2%82%A67%2C395%20%28%249.2%29,critically%20low%2C%20equating%20to%20%E2%82%A633%20%28%240.02%29%20per%20day.

[89] Alando C, Owoeye F, Oyugi M (2025). African roadmap towards 5% gdp on health by 2030. https://globesolute.com/2025/01/20/african-roadmap-towards-5-gdp-on-health-by-2030/#:~:text=This%20report%20evaluates%20the%20annual%20increases%20in%20domestic,GDP%20in%20total%20health%20expenditure%20%28THE%29%20by%202030.

