# Supplementary material for: Inequitable paediatric kidney transplantation in resource-limited countries: expert recommendations for Nigeria – a scoping review
Source: BMJ Glob Health. 2025 Dec 5;10(12):e017023. doi: 10.1136/bmjgh-2024-017023 (PMC12684210; doi:10.1136/bmjgh-2024-017023)
Supplement: online supplemental file 1 [file bmjgh-10-12-s001.docx]

**Supplemental Table 1. Examples of stringed keywords for databases search**

| **Database** | **Stringed keywords** |
| --- | --- |
| PubMed | ((((("challenge"[All Fields] OR "challenged"[All Fields] OR "challenges"[All Fields] OR "challenging"[All Fields] OR ("inequalities"[All Fields] OR "inequality"[All Fields] OR "inequities"[All Fields] OR "inequity"[All Fields]) OR ("access"[All Fields] OR "accessed"[All Fields] OR "accesses"[All Fields] OR "accessibilities"[All Fields] OR "accessibility"[All Fields] OR "accessible"[All Fields] OR "accessing"[All Fields]) OR ("prospect"[All Fields] OR "prospected"[All Fields] OR "prospecting"[All Fields] OR "prospection"[All Fields] OR "prospections"[All Fields] OR "prospectives"[All Fields] OR "prospects"[All Fields])) AND "renal transplantation"[All Fields]) OR "kidney transplantation in children"[All Fields] OR ("transplantability"[All Fields] OR "transplantable"[All Fields] OR "transplantated"[All Fields] OR "transplantating"[All Fields] OR "transplantation"[MeSH Terms] OR "transplantation"[All Fields] OR "transplantations"[All Fields] OR "transplanted"[All Fields] OR "transplanting"[All Fields] OR "transplantation"[MeSH Subheading] OR "transplantation s"[All Fields] OR "transplanter"[All Fields] OR "transplanters"[All Fields] OR "transplantion"[All Fields] OR "transplants"[MeSH Terms] OR "transplants"[All Fields] OR "transplant"[All Fields])) AND "developing countries"[All Fields]) OR "Sub-Saharan Africa"[All Fields] OR "low-income countries"[All Fields] OR "lower-middle income countries"[All Fields]) AND (english[Filter])  From 2003 to 2023 |
|  | TITLE-ABS-KEY ( challenges OR inequality OR access OR prospects AND "renal transplantation" OR "kidney transplantation in children" OR transplantation AND "developing countries" OR "Sub-Saharan Africa" OR "low-income countries" OR "lower-middle income countries" ) AND PUBYEAR > 2002 AND PUBYEAR < 2024 AND ( EXCLUDE ( DOCTYPE , "er" ) ) AND ( EXCLUDE ( LANGUAGE , "French" ) OR EXCLUDE ( LANGUAGE , "Spanish" ) OR EXCLUDE ( LANGUAGE , "German" ) OR EXCLUDE ( LANGUAGE , "Ukrainian" ) OR EXCLUDE ( LANGUAGE , "Portuguese" ) OR EXCLUDE ( LANGUAGE , "Polish" ) )  2003 to 2023 |
