# Supplementary material for: Inequitable paediatric kidney transplantation in resource-limited countries: expert recommendations for Nigeria – a scoping review
Source: BMJ Glob Health. 2025 Dec 5;10(12):e017023. doi: 10.1136/bmjgh-2024-017023 (PMC12684210; doi:10.1136/bmjgh-2024-017023)
Supplement: online supplemental file 2 [file bmjgh-10-12-s002.docx]

**Supplemental Table 2. Improving access to paediatric kidney transplantation in resource-limited countries**.

| **Reference** | **Populations/settings** | **Challenges** | **Solutions/Interventions** |
| --- | --- | --- | --- |
| Sakhuja et al.  2003 ^42^ | KT in India and  Pakistan. | **A.** Inadequate infrastructure and workforce: No reliable renal data. Only 5% have access to KT. Mostly living-related donation (30%- 40%). Disproportionately more female donors.  Cadaveric KT less than 2%.  **B**. High cost of KT: Donor commercialization. High cost of immunosuppressive drugs | **A.** Advocate for improved infrastructure for pre-emptive KT.  **B.** To lower the cost of KT, advocate for a health insurance scheme, produce dialysis machines and disposables locally, safe reuse of dialyzers and tubing, and use of generic immunosuppressive drugs to increase access. |
| Ghods and Savaj, 2006 ^43^ | KT in Iran between 1988 to 2005. | **A.** Altruistic supply of organs has been less than adequate, with severe organ shortage. Government engaged in regulated paid donations for living unrelated donors. | **A.** In 1988, Iran commenced a compensated program for living-unrelated kidney donations, significantly increasing KT.  By 1999, there was no more waiting list.  By the end of 2005, 19,609 transplants: 3,421 from living related donors, 15,356 from living-unrelated donors, and 823 from DDKT. The program mitigated ethical issues related to paid donations.  By 2005, Iran has no KT waiting lists, and over 50% of CKD-5 patients have functioning kidney grafts. |
| Chalise et al. 2010 ^44^ | KT in Nepal, August 2008. | **A**.Limited access to KT: Most KT is still done through medical tourism, which is expensive and involves illegal organ donation | **A**.Build a healthcare and professional transplant workforce that can support  KT programme. |
| Haddiya et al. 2012 ^45^ | This study reports a 10-year experience of KT in a Moroccan Hospital, and barriers to KT in Morocco. | **A**.Economic, sociocultural and legal constraints: The legal framework for LDKT was established in 1999, with only Rabat and Casablanca as approved centres. Currently, 37% of patients have medical insurance, despite KT being more cost-effective than haemodialysis.  **B**. Organ shortages: There is a significant shortage of living related donors, and discussing organ donation is often difficult. Cadaveric organ procurement faces challenges due to inconsistent emergency responses and family consent requirements, leading to refusals. Delays in establishing a public organ donation register and lack of resources for brain death diagnosis further complicate the situation.  **C**.Logistic and medical obstacles: There's a shortage of qualified staff and limited training due to new programs. Logistical issues include frequent medication shortages and lack of a dedicated facility at our university. Financial barriers complicate donations, as payment exemptions for pretransplant evaluations are applied inconsistently. These factors hinder progress in KT.  **D**. Difficulty of recruitment: Recruiting patients for KT is difficult, with 97% of dialysis patients not on waiting lists. Key reasons include 13% refusal, 14% medical issues, and 70% lacking a related donor. Additionally, there is a lack of awareness about the benefits of transplantation in Morocco. | **A/C/D**. Enhancing awareness is crucial to boost transplant rates. The Ministry of Health should promote KT as a cost-effective alternative to haemodialysis for CKD-5.  **B**. Promoting awareness through media, schools, and mosques can help build public trust in the donation process, making it easier to procure kidneys from cadaveric donors. |
| Arogundade  2013 ^24^ | KT in Nigeria,  between 2000 and  2010. | **A**. A significant barrier is the inadequate funding of KT. Poor access because of OOP expenditure by poor patients.  **B**. Donor shortage: Living donors are scarce. Deceased donation not available.  **C**. Inadequate laboratory support: Tissue typing/cross-matching, drug level monitoring, and some viral studies are done overseas.  **D**. The need to import antiviral drugs and induction agents. This partly explains the increase in mortality from Cytomegalovirus infection.  **E.** Low volume of transplantation. Proficiency is retarded because of low KT rates. | **A**. Enhance access to KT through adequate health insurance. Hasten to enact relevant KT legislation to  increase access and prevent organ commercialism and trafficking.  **B**. Cadaveric donation would alleviate organ shortage.  **C**. Local tissue-typing and drug levels  monitoring laboratories will reduce  costs.  **D.** The author proposes the  establishment of three publicly owned KT centres. |
| Rizvi et al. 2013  ^46^ | PKT in Pakistan in  the 1980s. The  model of community-government partnership has been sustained  for over four decades with increasing support from the government  and community.  The government assistance for infrastructure, equipment, and up to 50% of the operating budget, whereas in the community, including affluent individuals, corporations donate the remaining 50%. ^75, 76^ | **A. Organ shortage:** Social issues/Poor donor pool: People unwilling to donate kidneys. Potential  donors are afraid of complications. Organ shortage from lack of deceased donation.  **B**. Economic issues: Public funding of KT  competes with other healthcare needs. The  cost of immunosuppressants is exorbitant.  **C**. High infections pose a threat via poor  allograft survival.  **D.** Logistics issues: Transplant centres are  distant from where patients live, thereby  incurring more cumulative costs | The Pakistani KT programme anchors  on equity, transparency, accountability,  and accessibility regardless of  socioeconomic and cultural  diversities. It provides a one-stop  hospital where all needed transplant  services are available.  **A**. Social acceptance of KT increases organ donations  **B.** Cost-saving strategies, such as self-sufficiency in KT consumables, including in-country production of  dialysate, use of simple cheap hemodialyzers, use of generic calcineurin inhibitors, and pre-emptive  arterio-venous fistula formation.  **C**. The capacity to diagnose infection quickly was developed.  **D**. KT services at the doorsteps of patients, and costs of travel and accommodation are reimbursed.  Immunosuppressants are provided free  for life |
| Kute et al. 2014 ^47^ | In India, access to LDKT is limited because of HLA desensitization and ABO-incompatible due to high costs and increased infection risks from intensive immunosuppression. Kidney paired donation (KPD) offers a solution | **A**. Issues regarding legal permission in India  **B**. Concerns regarding the donor-recipient age difference affecting the allograft outcome.  **C**. Concerns about difference in graft survival between KPD *versus* LDKT?  **D**. Concerns whether increased cold ischemia time (CIT) would affect the allograft outcomes?  **E**. Waiting time for deceased donor *versus* KPD transplantation/LDKT  **F**. Should KPD be performed for better human leukocyte antigen (HLA) matching? | **B/C**. Age differences should not hinder KPD, as recipients can succeed with donors up to 30 years older. KPD reduces waiting times and dialysis costs.  **D**. Prolonged cold ischemia times (over two hours) did not negatively affect outcomes, indicating that transporting living donor kidneys over longer distances is viable.  **E.** To improve KT, we should expand KPD to multicentre chains, allowing for the shipping of living donor organs without compromising outcomes when CIT is under eight hours.  **F**. Patients with CKD-5 and compatible, fully HLA mismatched donors over 45, especially O blood group donors, should be included in KPD programs, as this can nearly double match rates for incompatible pairs. Future strategies should include regional and national exchanges, list exchanges, three-way donations, domino chains, and virtual crossmatch for highly sensitized recipients. A regional KPD pilot program should precede a national program. |
| Ackoundou-N’Guessan et  al. 2015 ^48^ | KT in Ivory Coast  between 2012-2014 | **A.**Lack of adequate legislation to support  KT/Organ donation.  **B**.Lack of laboratory capacity to support  deceased donations.  **C**.Limited infrastructure to monitor blood  levels of immunosuppressants.  Scarce and expensive antiviral agents and  Immunosuppressants because they cannot be produced in Ivory Coast | **A**.Engage in more public campaigns to  increase awareness for living donations.  Government to encourage organ donation by incentivizing donors and  provide financial support.  **B/C**.The government should provide well equipped KT centres. To adapt the private-public partnership model of Pakistan. ^46^ |
| El-Nono et al.  2015 ^49^ | KT in Yemen between 1998-2012 | **A**.Limited resources and medical workforce, and lack of capacity to support DDKT.  **B**.Poor access to immunosuppressants, lack of facilities for tissue typing and blood assays for immunosuppressants. | **A**.Establishment of KT centres staffed by well-trained personnel.  To expand transplantation to include cadaveric KT and liver transplantation. |
| Ulasi and  Ijoma, 2016 ^22^ | The report covers KT  in Nigeria from 2000  to 2014 | **A**. Weak infrastructure and financial support: Lack of capacity for tissue typing  and blood drug levels monitoring. No health insurance to cover the cost of transplantation. No deceased donor program.  **B.** Medical tourism: Nigerians engage in  medical tourism, lack of faith and mistrust in local KT programmes.  **C**. Lack of legislation and national registry for donation, transplantation, and regulations.  **D**. Poor organ pool: Sociocultural and religious beliefs and practices encumber both living and deceased organ donation.  Only living donation is being done in Nigeria currently.  **E**. Exorbitant cost of transplantation and immunosuppressants via OOP health expenditure. | **B**. Four fully equipped  centres may suffice for transplantation  in the country's four main regions.  **C/D**. There is need to strengthen the country’s Health Bill to regulate organ  donation and prohibit exploitative organ donation and unscrupulous medical tourism and commercialization.  **E**. Advocate for generic or subsidized immunosuppressants to save costs and make RT more accessible. |
| Abraham et al. 2016 ^50^ | Expanding DDKT in India and a proposed model for other developing countries | **A**.The main barrier to DDKT in India is a shortage of harvested organ pool. | **A**.Success is dependent on Government hospitals providing free or subsidized LDKT for underprivileged populations. A Government hospitals' free or subsidized LDKT is vital for underprivileged populations. Progress in DDKT accelerated in Tamil Nadu since 2008. The Multi Organ Harvesting and  Networking (MOHAN) Foundation’s grief counsellors help boost organ donation, achieving 65% consent rates by training 813 transplant coordinators since 2010. The counsellors have significantly improved organ donation rates, achieving up to 65% consent when they counsel grieving families, emphasizing the importance of effective support in boosting donations. |
| Okafor 2016 ^29^ | KT in a public  hospital in Enugu,  between 2010 and  2014. | **A**. Delays in KT caused by financial constraints, lack of transplant expertise,  and shortage of organs. Most KT  recipients had their transplants in India.  Gender disparity that favors men.  **B**. Financial constraints delayed transplants in 66% of patients. Non availability of donors was reported in 17.2%. Acquisition was made through OOP expenditure, with no health insurance.  **C**. Deceased donation is not available.  Poor follow-up in 60% of patients who rely on telemedicine for overseas  consultation.  **D**. Challenges to improving KT in Nigeria  include rampant medical tourism and lack  of regulatory policy | **A/B/C/D**.The expansion of KT in Nigeria requires the collective efforts of all  stakeholders, including the  government, medical professionals, and  the general population. |
| Abderrahim et al. 2017 ^51^ | The study report the  results of 30 years of experience at the first KT centre in Tunisia, between June 1986 and June  2016. | **A**. Lack of supportive legislations: Before June 1986, around 120 Tunisians with CKD-5 received KT abroad. A legal framework for organ transplantation was established in Tunisia in 1991, allowing only public hospitals to perform transplants, with the government covering costs. Despite these measures, kidney transplant rates remain low, with only 10% of eligible dialysis patients on waiting lists.  **B**. Organ shortage because of political instability, under-reporting of brain deaths, and family refusals. Tunisia leads the Arab world in kidney transplants, but only 20% of patients receive kidneys from deceased donors, often due to gender imbalances and insufficient public awareness.  **C.** Complications like early graft loss and infections highlight the need for improved strategies to enhance transplant outcomes. | **A/B/C.** Provision of supportive legislations, increasing organ donations, improving renal transplant capacity have increased access to KT in Tunisia. The Tunisian KT activity which started in June 1986, at Charles Nicolle Hospital in the capital city of Tunis had resulted in 1686 transplants by December 2015, performed in the 6 transplant centres in Tunisia. |
| Amira et al.  2017 ^25^ | KT in a government  funded public  hospital in Lagos,  Nigeria, January  2012 to December 2014 | **A**.Organ shortage because of  ever‑increasing burden CKD-5, unwillingness towards live donation, decrease in the supply of organs, and  failure of transplanted kidneys.  **B**. Poor government funding and the exorbitant cost of OOP expenditure for KT.  **C**. Transplant tourism in Nigeria is driven by a lack of confidence in local technical  expertise and cheaper options in some Asian countries, leading to significant business transactions. | **A**. To increase the donor pool, expand donor criteria, and organize paired exchanges of kidneys. Address cultural  and religious biases against donation with public enlightenment campaigns.  Legislation and improved infrastructure  are also needed to support deceased  organ donation.  **B**. Advocacy to improve government funding and intervention by health  insurance agencies. Consider the Rizvi  model above. ^46^  **C**. Raise public awareness about the availability of KT in Nigeria among the  populace. |
| Sethi et al. 2017  ^52^ | Survey of PKT in  India, 2017 | **A.** Challenges to improving access to transplantation in some areas include limited access to tertiary-level healthcare and transplant centres, no program for paediatric HD,  **B**. Lack of transplant workforce and expertise, lack of a renal registry and wait-list, lack of supportive health insurance, lack of capacity for deceased donation, and lack of living related donors.  **C.** Organ shortage. Deceased donation is challenged by a lack of requisite facility and  infrastructure, No capacity for pre-emptive transplant.  **D**. Dearth of a renal registry and waiting list.  **E.** Regional variability in access to KT that parallels different socio-economic  development in India. | **A**. Enhance the ability to diagnose CKD/CKD-5 promptly in children,  Good referral system between general physicians and nephrologists will be  helpful.  **B/C/D/E**. Increasing access to KT must include legislation to support the provision of infrastructure, workforce and financial resources needed to expand KT in India. |
| Popoola et al.  2018 ^26^ | Expanding KT organ  donor pool in Nigeria,  2018 | **A**. Dearth of transplant facilities in poorly equipped health centres. Uneven distribution of HD centres across national  regions, weak transplant expertise, poor hospital infrastructure to support KT, poor government-backed funding for KT.  **B**. Unwholesome medical tourism to India or Pakistan still a common finding with its attendant unwholesome  commercialization and organ trafficking  **C**. Scarcity of donated organs remains a significant constraining factor | **A**. Amend the Nigerian Health Bill 2014 to allow for donors’ compensation. Need to have a kidney registry. have a specific definition of  brain-death and proper legislation to regulate transplant activities and  administration.  **B.** Intensify public campaigns on CKD-  5, the safety of living donation, and KT. Avoid duplication of efforts and  resources by hosting a renal registry on existing national data platforms  **C**. Development of regional transplant centres with all the supportive facilities  that can support both living and deceased donations. Collaborate with the Federal Road Safety Corps (FRSC) as an important  stakeholder in deceased organ donation. Save costs by encouraging domestic production of immunosuppressants. |
| Chironda et al. 2019 ^53^ | Authors highlight the benefits, challenges and recommendations to provide future directions for KRT in Rwanda | **A.** Policies and guidelines. There is limited expansion of coverage of medical procedures related to KT. Weak legislation does not encourage organ donation.  **B.** Accessibility. Most Rwanda’s population is in a rural setting, affected by distances as the renal centres are situated in the  Cities.  **C.** Shortage of nephrologists and nephrology nurses  **D**. Financial burden as well prevent access.  **E.** Community education. Kidney rejection is very common due to lack of education. Post-transplant infections like tuberculosis, nocardiosis and many others that the communities need  to be aware of and may require urgent attention if they occur or present. | **C.** Empowering and equipping the existing health care settings with  dialysis units with adequate supplies and human resources are imperative. Training of workforce is important.  **E.** University of Rwanda, School of Nursing with African Health Care Network, Rwanda Biomedical Centre and Rwanda Kidney Organization have been involved in community outreach programs relating to prevention and screening of CKD. |
| Bakr et al. 2020 ^54^ | Reports of KT at a centre in Egypt since 1976. | **A**.Deceased donor transplant shortages are a significant issue in Mansoura, Egypt, and other Arab countries. A controversial incident in the early 1990s, involving organs from executed criminals, led to a ban and increased distrust in organ donation. | **A**.Efforts to promote deceased donation, including legal frameworks and awareness campaigns, have not significantly boosted public support. Building community trust is essential to address this challenge. |
| Kute et al. 2020  ^55^ | Deceased organ  donation (including  kidney) and  transplant in India,  2013-2018 | **A**. DDKT encumbered by ignorance, religious beliefs, and mistrust in the  system.  **B**. Public misconceptions about deceased  donation: Negative media publicity in movies projects organ trafficking as a  norm | **A/B.** To improve access to organ donation, public education, healthcare  worker education, and family support are essential. Collaboration, grief counselling, non-financial incentives, ethical regulation, and regular audits through a renal registry are important. |
| Saeed 2020 ^56^ | KT in Syria between  1990-2018 | **A**.Only living-related and non-related living donations are possible. Cadaveric donation is not in place. Ignorance and religious beliefs hinder deceased and living donation. Also, mistrust of the system based on witnessed experience.  **B**. Shortage of transplant physicians and surgeons because of brain drain following the Syrian war. Immunosuppressants have become scarce because of the war.  **C.** Financial constraints and shortage of  transplant workforce and donated organs. | **A**.Increase the country’s pool of organs available for transplantation by encouraging deceased donation.  **B/C.** Address all the problems noted |
| Pais et al. 2021  ^57^ | PKT in India, 2013-  2018 | **A**. Children on the organ waiting list should be prioritized. Unrelated living  donation is scarce without consideration for financial gain.  **B**. Follow-up is hampered by OOP expenditure  for travel and lodging.  **C**. Parental refusal  of transplantation is another obstacle to the success of KT. Fathers are usually  unwilling to donate kidneys.  **D**. Lack of deceased donation is common, limiting the organ pool available for transplantation.  **E**. Unwillingness to be a  living donor because of ignorance and misconceptions about donation is  common. | **B**. Reduction of LTFU:  Families need both government and private financial support for  transplants. Achieving UHC is crucial for equity in transplants, especially for  CKD-5 patients. Overcoming distance as a barrier by expanding paediatric nephrology outreach clinics. Good  referral system and professional networking is crucial for patients’ follow-up. Reducing indirect losses to families by liaising with caregivers and donor employers for paid medical  leave.  **C.** Education about CKD and awareness of transplants: This will promote earlier diagnosis and treatment  **E.** Increasing donor availability: Place  emphasis on children with CKD-5 in  deceased donation. International collaboration and development of Sister Transplant Programme is beneficial |
| Eke et al. 2021  ^21^ | PKT within and  outside Nigeria, 1986  to 2019 | **A.** The high cost of KT: The lack of a national program for renal care services  such as dialysis and KT is a major challenge in Nigeria. Poor government funding is multifactorial. Despite additional expenses, KT in India is still more affordable than in Nigeria.  **B**. Donor organ shortage. No program for cadaveric transplants. Preference for male children with CKD-5 to have access to KT.  Parents, especially fathers, are reluctant to  donate kidneys.  **C.** Unavailability and high cost of immunosuppressants. The need to import immunosuppressants makes them rather  expensive.  **D**. Exorbitant cost of measuring blood  levels of immunosuppressants contributes  to poor outcomes.  **E.** Dearth of transplant facilities and manpower.  **F.** Lack of workforce: Nigerian paediatric  nephrologists provide perioperative care  for children transplanted overseas.  Prolonged HD before transplantation can be detrimental due to the need to treat donor-specific antibodies. Improved  access to transplantation and donor organs in Nigeria is needed to minimize  the negative impact on patient outcomes.  **G**. Because of financial constraints, patients are under-dialyzed with 1–2  times weekly haemodialysis. Chronic ambulatory dialysis is not available in  Nigeria because of the cost as consumables need to be imported. | **A**. To improve access to KT care, the federal government can take several  steps. Firstly, they can encourage in country production of KT consumables  and save costs. The federal government can take over the cost of KT. To raise  public awareness and education, advocacy campaigns for KT are  necessary.  **B.** Advocacy campaigns to kickstart deceased organ donation: Developing  effective harvesting programs and referral systems are important for  improving access to KT care in Nigeria. |

CKD=chronic kidney disease, ESKD=End Stage Kidney Disease, KT=kidney transplantation,

PKT=Paediatric KT, KRT=Kidney Replacement Therapy, OOP=out-of-pocket, ISN=International

Society of Nephrology, TTS= The Transplantation Society, IPNA= International Paediatric Nephrology

Association, ISPD= International Society of Peritoneal Dialysis, IPTA= International Paediatric

Transplant Association (IPTA), HIC= high income countries, LMICs= low-and middle-income countries,

LIC=low-income countries, UHC=universal health coverage, LLMIC=low-and low-middle income

countries, NAN= Nigerian Association of Nephrology, PNAN= Paediatric Nephrology Association of

Nigeria, TAN= Transplant Association of Nigeria, MMF=mycophenolate mofetil, CNIs= calcineurin

inhibitors, LDKT=living donation kidney transplantation, DDKT=deceased donation kidney

transplantation, HLA=human leucocyte antigen, WHO=World Health Organization, LTFU=loss to follow

up, PD=peritoneal dialysis, HD=haemodialysis
