# Supplementary material for: Inequitable paediatric kidney transplantation in resource-limited countries: expert recommendations for Nigeria – a scoping review
Source: BMJ Glob Health. 2025 Dec 5;10(12):e017023. doi: 10.1136/bmjgh-2024-017023 (PMC12684210; doi:10.1136/bmjgh-2024-017023)
Supplement: online supplemental file 3 [file bmjgh-10-12-s003.docx]

**Supplemental Table 3. Experts’ opinions on how to improve access to paediatric kidney transplantation in the world's resource-limited countries.**

| **Reference** | **Populations/Settings** | **Challenges** | **Solutions/Interventions** |
| --- | --- | --- | --- |
| Persy et al. 2010 ^58^ | KT in Africa, Bamako, Mali,  December 4-6, 2008 | **A**. High costs of KT: In Sub-Saharan Africa, KT is mainly funded by out  of-pocket expenditure. Barely 4% continue dialysis for more than 3  months, and KT was performed in only nine African countries in 2008.  **B**. Inadequate donor pool: Only living donation is possible in most emerging  countries, with South Africa and Tunisia being exceptions utilizing deceased donors. Poor donor availability because of ignorance, culture, and superstitions. Brain-dead  needs to be well defined before DDKT can be enhanced.  **C.** Weak national health systems: No renal registry in most developing countries. Also lacking is human capacity to keep records.  **D.** Prevailing endemic infections: Most  post-renal transplantation deaths are from infections. | **A**. Strategies to reduce the costs of immunosuppression in KT include using azathioprine instead of MMF, generic CNIs, and co-administering CNIs with drugs that increase  bioavailability.  **B**. Preventing organ  trafficking and exploitative renal transplantation requires renal  registries, KT legislation, and public education to improve organ donation  consent rates.  **C/D**. International  collaborations can also help improve access to KT and strengthen healthcare  systems, and pre-transplant vaccination and chemoprophylaxis  should be encouraged to prevent post-transplant infectious complications. |
| Akoh, 2011 ^59^ | KT in developing  countries in 2011 | **A.** No receipt of KT because KT centres were few, lack of transplant expertise, and unsafe with records of poor outcomes.  **B**. Organ pool restricted to living donation alone.  **C.** Transplant tourism and its attendant organ trafficking and unwholesome commodification of  human organs.  **D.** Poor government funding: Poor  commitment to health expenditure  because of bad economies.  **E.** Poor public attitude towards living  donation because of ignorance,  mistrust, and misconceptions.  **F.** Dearth of organ pool. | **A**. Improve regulatory legislation  guiding kidney transplantation.  **B.** Starting with LDKT and then progressing to unrelated donation is a  good strategy. DDKT can come later in well-equipped tertiary or teaching  hospitals with existing facilities.  **E.** Health and public education initiatives and legislation on brain death will improve access to DDKT. |
| Spearman and  McCulloch, 2014 ^60^ | Solid organ paediatric  transplantation (  heart, lungs, kidney,  pancreas, liver, and  intestine) in Africa,  2014 | **A**. Lack of centralization of transplant centres disperses scarce transplant workforce and prevents proficiency  from developing because of inadequate experience that comes with low transplant rates.  **B**. Problem of transition of teenagers to the adult transplant program.  **C**. Transplant assessment: In many African countries, the high burden of infectious diseases may prevent a child from undergoing transplantation. Infections also  impact allograft outcomes.  **D.** Post-transplant care: No organized follow-up care, unavailability of  immunosuppressants, and limited capacity to monitor their blood levels on a long-term basis.  **E.** Inadequate donor pool because deceased donation is encumbered by many factors, including a lack of legislation, diagnosis of brain death, and ignorance and misconceptions.  Infrastructure for deceased donation is also not well-developed.  **F.** Transplant tourism Pervasive poverty enables transplant tourism and its attendant organ trafficking and the unwholesome commodification of human organs. | **C**. International professional  organizations like IPNA and ISN should continue to provide fellowship  training programs, especially for KT science, for 1-2 years.  **E.** It is essential for national  governments and the international transplant community to lobby  pharmaceutical companies to make immunosuppressants available and affordable in Africa.  **F.** Legal and cultural recognition of  brain death, and establishing organ procurement committees, are crucial  for transparency and documentation of  KT activity. To promote  transplantation, establish donor and recipient registries and educate the  public about organ donation. Begin with a renal dialysis program and ensure necessary infrastructure before starting KT activities. Start with adult KT before  implementing deceased donation and PKT programmes. |
| Muralidharan and White, 2015 ^61^ | Review current data  on the global burden of end-stage kidney disease and the distribution of major risk factors and compare this to access to KT in LMIC in 2012. | **A**. Health systems factors, such as cost constraints and limited technical capacity, hinder transplantation services in Tunisia.  **B**. Cultural attitudes towards organ donation and the effectiveness of existing legislation are critical to increasing donor rates. Only 20% of kidney transplants in Tunisia come from deceased donors, often due to gender imbalances and low public awareness.  **C.** Insufficient infrastructure, poor system coordination, and a shortage of transplant surgeons are major barriers to developing organ transplantation services. | **A**.Countries with large dialysis populations and public funding need more KTs. The Spanish organ donation model successfully centralizes coordination and utilizes transplant coordinators to boost organ recovery. ^77^ In countries like Pakistan, where dialysis and transplant costs aren't covered by insurance, innovative financing is essential. ^46^ The Sindh Institute of Urology and Transplantation offers free LDKT and lifelong immunosuppression through community-government partnerships, removing out-of-pocket expenses. ^46^  **B**. Legislation must also permit brain death declarations and organ removal for transplantation. Despite Tunisia’s legal framework established in 1991 to address organ shortages, challenges like political instability, under-reporting of brain deaths, and family refusals persist. Additionally, issues like early graft loss and infections point to the need for better strategies to improve transplant outcomes. |
| Muller 2016 ^62^ | KT in developing  countries of Africa,  2016 | **A**. LDKT is available in South Africa, Tunisia, and Sudan, but DDKT only  available in South Africa. Deceased donation is limited by a lack of supportive hospital infrastructure and an appropriate legislative framework.  **B.** Exorbitant charges for transplant surgery, post-operative care, and maintenance immunosuppression.  **C.** Disparities in the distribution of  dialysis and transplant centres in urban areas tend to affect treatment.  **D**. Challenges to improving access to transplantation in Africa include the  unavailability of transplant  workforce, lack of transplant hospital infrastructure, and a trust deficit in the health system. | **A.** To improve transplantation outcomes, it is recommended to  establish a successful living donor program before implementing deceased donation. Advocating for  funding, infrastructure, and renal registries can help achieve this.  Additionally, dispersing  transplantation activities across a country's geographical locations can  also contribute to better access and outcomes for patients in need.  **B.** To improve transplantation access  in Africa, estimate the CKD-5 burden and lobby for cheaper  immunosuppression. Governments must allocate resources and establish  legislation for transplantation. A Pan  African database can help negotiate cheaper immunosuppression.  Address the brain drain from Africa. |
| O’Connell et al.  2020 ^63^ | ISN’s GKH  Conference United  Arab Emirates, 2018. | **A.** Legislation and regulation: Legislation needs to be improved in  some regions of Asia and Africa. Deceased donation lacks supportive legislation.  **B.** Inadequate government-backed funding of transplant: Lack of a health insurance scheme to finance  transplantation. Public funding is limited, occurring in only 49% of  countries, with higher rates in high income countries.  **C.** Health workforce: In Africa, the Middle East, South-east Asia, Central  Asia, and Oceania, there is a lack of transplant expertise.  **D**. Inadequate social support for transplantation in society: There exists a gap in patients' and the community's understanding of KT, hence significant anxieties and fear of  organ donation.  **E.** Unavailability of renal registries in most parts of Africa, Asia, and Eastern Europe.  **F.** Deceased donation is not well established. Promote deceased donation through proper legislation and the provision of medical  infrastructure. Address ignorance and misconceptions surrounding deceased donation.  **G**. Ethical standards: Develop standard legislation that promotes ethical living donation and LDKT. | **A.** Promote deceased donation through proper legislation and provision of medical infrastructure.  Address ignorance and  misconceptions surrounding deceased donation.  **B**. Health financing and  insurance: Public funding models for KT include dedicated funds through  taxation, health insurance, and charity  schemes.  **C.** Health workforce: Running a KT program requires a specialist workforce, and a national public program should be implemented at a  tertiary hospital.  **D**. Patient engagement and community education are needed to promote deceased organ donation and transparency in resource allocation.  **E**. Registries: The KT registry is crucial for monitoring KT quality.  Collaborative Transplant Registry offers free software to document healthcare delivery and evaluate KT outcomes.  **F.** Promotion of deceased donation. Good legislation will promote deceased donation.  **G.** Development of ethical standards: To promote safe and accessible renal  transplantation in under-resourced countries, it is crucial to focus on  optimizing consent and care for both donors and recipients, ensuring  thorough certification of donor suitability, criminalizing trafficking,  ensuring financial neutrality and covering all donation costs. One program that is working towards these objectives is the ISN-TTS Sister Transplant Centres, which aims to connect resource-limited countries with established centres in high income countries. |
| Loua et al. 2020 ^65^ | WHA and the United  Nations General  Assembly resolutions  on organ donations  and transplants in 47  countries in the WHO  African Region, 2020 | **A.** Dearth of information on organ donation and transplantation because  supportive registry and legislation are not in place.  **B.** Lack of access to transplantation centres: There is a lack of human  sources and advanced technology.  **C**. Inadequate financing for organ transplant programmes.  **D**. Unavailability of  immunosuppressive agents. | **A**. Establish legal and regulatory frameworks:  **C.** Provide good funding for KT programme via public, private, and public-private initiatives. |
| Arogundade et al. 2021 ^64^ | Experts’ opinions on burden of CDK-5 and rarity of KT in Sub-Saharan Africa | **A**.Funding and donor shortage are severe  mitigating factors for KT.  **B**. Most countries do not have an effective renal  transplant policy, enabling legislation and cadaveric donation is still largely unavailable.  **C**. The lack of incentive for donors has been  postulated to be another factor affecting organ donation. | **A** detailed study of the Ira  nian model, where a state-sponsored monetary compensation has virtually eliminated the long waiting time for transplants, may be  explored as it may eliminate unethical commercial donation. ^43^ |
| Esezobor et al. 2021  ^66^ | Paediatric  nephrology,  including PKT in  Africa, 2021 | **A.** Lack of or limited capacity for KT. It is a common practice for transplant tourism.  **B.** Limited pool of donor kidneys as DDKT is still uncommon because of non-existent legislation for brain  death.  **C**. Inadequate diagnostic modalities needed for pre-transplant assessment.  **D**. Scarce and expensive  immunosuppressants. Concerns about  formulations that affect paediatric populations.  **E.** Inequitable distribution of scarce paediatric nephrology personnel. Brain  drain is a significant problem in Africa.  **F**. Renal care is expensive and out-of-reach for most Africans because it lacks government funding and social insurance financing | **B.** Make legislation available.  **E**. Training of allied health  professionals and general physicians need to support renal care services. |
| Elrggal et al. 2021 ^67^ | ISN Fellows and  country representatives from developing countries | **A**. Cultural, religious and educational barriers  **B**. Organ shortage and prolonged waiting times  **C**. Transplant tourism, organ trafficking, legal challengers  **D.** Financial and organizational barriers | **A**. Education and awareness initiatives are crucial for increasing public acceptance of live and deceased organ donations. It's important to address misconceptions about deceased donation and engage policymakers to support comprehensive KT programs. Many countries need to clarify the concept of brain death and implement legislation for deceased donation.  **B**. Expanding the donor pool can be achieved by promoting paired or pooled kidney donation in countries with established transplant activities.  **C**. Strict regulations against organ trafficking are necessary, with governments enforcing inspection measures and penalties.  **D**. Additionally, improving healthcare funding to cover transplant costs is essential for national programs. Countries should identify and rectify infrastructural issues in collaboration with transplant professionals and established programs, such as sister centre projects and fellowship training for nephrologists from developing nations. |
| Okpechi et al. 2022  ^68^ | Kidney care by the  ISN–GKH Atlas  Africa data, 2018 | **A**. Enormous shortage of skilled renal care workforce.  **B**. Shortage of funding and exorbitant OOP payment.  **C**. Shortage of human  and technical resources to support transplant programs.  **D.** Lack of supportive regulatory and authorizing legislative frameworks. | **A**. Compensate for inadequate  professional workforce by shifting and sharing responsibilities among allied health professionals. The supportive roles of AFRAN in training and  mentoring cannot be  overemphasized.  **B**. Make concerted  efforts to reduce out-of-pocket health expenditure by adopting the WHO's  Global Action Plan on CKD  management.  **C.** Strengthen existing living-donor KT centres or establish new KT centres if  well, supported.  **D**. The is an urgent need to have a  renal registry across countries and  regions of Africa. |
| Bamgboye et al.  2022 ^69^ | KT in Mexico, India,  Nigeria, Ghana, and  Zimbabwe, and PKT  in Africa, 2022 | **A**. Limited capacity for KT limited to private hospitals. Payment for KT in sub-Saharan Africa is not covered by insurance. Out of the eight countries that have KT programs, most perform LDKT.  Morocco, Algeria, Tunisia, and South Africa have a DDKT programme.  Nigeria: Has the capacity to perform LDKT. However, most KT take place in private centres, located in urban cities, accounting for more than 90% of transplants.  **B**. Dearth of government financing of KT: Payment for KT and other forms of KRT in many African countries remains a catastrophic OOP health  expenditure, with exceptions being South Africa, Mauritius, and Kenya. Cote d’Ivoire, Nigeria, Angola, and Uganda have supportive regulatory legislation.  **C.** Lack of adequate legislative framework to support KT in most developing economies  **D**. In Africa, the late diagnosis of children with CKD hinders management and care. Children and  their mothers suffer most because paediatric nephrology is rudimentary,  and females rarely receive renal transplants.  **E.** No deceased donations in LLMICs because legislation to support it is lacking.  **F.** The coronavirus disease-2019 erodes earlier gains of KT in Nigeria | **A**.Concerted efforts by IPNA, the ISN, the ISPD, and the IPTA in the international training and building of  manpower cannot be overemphasized and must be sustained. |
| Saeed, 2022 ^70^ | The author provided  an overview of adult  KT and PKT in  Middle East (ME)  countries, 2019-2021 | **A**.Pre-transplant challenges:  The cause is unknown, but it is prevalent in some regions. KT care is challenging due to comorbidities and  limited facilities. DDKT for children are limited in most Middle Eastern countries due to size disparity, lack of  legal framework, and public  awareness.  **B**.Post-transplant challenges: Medication issues include unavailability, high costs of  immunosuppressants, and  immunosuppressive toxicity. Acute rejection rates are high but decreasing  with the introduction of effective immunosuppression.  Minimizing immunosuppression  strategy is still rare as concerns remain about its safety.  Infections are common in children and are responsible for poor allograft function and patients’ outcomes.  Also, serious concerns about  inadequate growth and development in children after KT. | **A/B**.The author advocates for the  community-government funding partnership in Pakistan as earlier  described by Rizvi et al. 13 above. |
| Iyengar and  McCulloch, 2022 ^71^ | PKT in under-  resourced regions of  the world in 2022 | **A.** Lack of renal registries in some countries.  **B.** Medical issues: PKT in LLMIC faces medical issues such as recurrent native kidney diseases, genetic  testing, and combined kidney-liver transplantation.  **C**. Lack of capacity and health workforce: In most centres, PKT is performed by adult nephrologists  (61%) in private hospitals, although most PKT occurs in children weighing 20 kg and above. Medical tourism is also common in some LLMIC centres.  **D**. Poor financing of KT programmes and services: The most crucial challenge to KT in LLMIC is the  unaffordability of KT because of the high costs. Families face OOP  catastrophic health expenditure, and there is little or no government support or support from health insurance.  **E**. Barriers beyond financial  handicap: Uneven distribution of KT  programmes and renal care services  hinder equal access to KT. There is also the problem of social rejection of KT because of ignorance,  mistrust, and misconceptions.  **F**. Ethical issues: Disparity in access constitutes a problem of unethical  inequalities in LLMIC. | **A**. There is a need to finance and make adequate legislation to support KT in  under-resourced countries of the world.  **C**. To enhance workforce capacity, training nurses in KT science can be a useful strategy. Making PKT training mandatory in post-doctoral medical curricula is also crucial.  **D**. A hybrid public-private  partnership model of financing KT  that is successful in Pakistan can also be adopted in under-resourced countries of the world.^46^ |
| Mudiayi et al. 2022  ^72^ | ISN-GKH Atlas  survey, which  addressed KT across  various countries and  regions, 2018 | **A**. LICs often lack the KT facilities, renal registries, skilled personnel, expertise, and finance and funding to  back up KT programmes.  **B**. Nigeria, Ghana, Kenya, Sudan, Morocco, Tunisia, and Algeria have some KT capabilities but low incidence rates. South Africa and Sudan have substantial annual  numbers of transplants.  **C.** No DDKT: Cause is multifactorial in LICs.  **D.** Low pre-emptive KT in LICs: LICs often lack the necessary capacity and expertise to engage in  pre-emptive KT.  **E.** In LICs, the absence of renal registries restricts KT.  **F.** Limited government funding in LICs often leads to catastrophic OOP  health expenditure that induces household medical poverty | **B/C.** Encourage governments of LICs  to increase the capacity and facility for DDKT. Increase awareness campaigns  to enhance the availability of KT.  **E**. The WHO recommends KT as part of UHC. Where this is not feasible,  public-private partnerships have proven successful in improving access  to healthcare services and KT care in countries like Pakistan, Iran, Kenya,  and the Philippines |
| Roberts et al. 2023  ^73^ | The Transplant Link  Community (TLC)  summarizes the  challenges and  barriers experienced  in developing PKT  services in LMICs  over the past 15 years | **A**. Lack of local government funding and will to develop KT services.  **B**. Inadequate KT infrastructure and workforce including lack of paediatric nephrology  medical, nursing, and allied  professional clinicians, turnover of trained key staff.  Variable availability of  immunosuppression and monitoring. Infrastructure for clinical data collection not readily available.  **C**. Reliable supplies of medication and surgical equipment.  **D.** Inconsistent political support.  **E**. Social issues, clinic non-attendance, and non-concordance with  medication | **A/B/C/D/E**. The support of the TLC can be expanded to include advocating for the countries’ governments to provide  financial and legislative support to KT programmes in LMICs. |
| Vo et al. 2024 ^74^ | Experts’ opinions of  IPTA on how to  develop and expand  renal and liver  transplant in LMICs | **A.** Difficult to reach centres  because of geographical locations of transplant centres  **B.** Socioeconomic disparities limiting access to transplant and long-term immunosuppressive therapy  **C**. Budget constraints, insufficient knowledge, technology, necessary  infrastructure, and logistics.  **D**. Health system structure that gives priorities to private or public.  **E**. Organ shortage from various reasons including the lack of infrastructure,  dedicated organizations, institutional mechanisms, notification on potential  brain-dead donors, and adequate donor care hinders the efficient and effective organ donation process.  Furthermore, the lack of public awareness, understanding of brain death and organ transplant benefits, lack of education and support among the public and medical profession, and cultural and religious beliefs also contribute to organ shortage.  Additionally, mistrusts of medical professionals abound.  **F.** Lack of supportive legal and government support | **A.** Charities, financial aid programs, and government funding can all contribute to increase access to transplant centres.  **B.** Charities, financial aid programs, non-governmental philanthropic and government funding can assist in transplant surgery and cost of medication. Pharmaceutical companies to produce subsidized  immunosuppressants or to produce low-cost  generic immunosuppressants.  **C.** Cooperation with seasoned transplant centres to provide support  and training. Liaison with public and private sectors and governmental and  non-governmental groups  **D.** Form local partnerships and local government funding; private sector  investment in the field of  transplantation; collaborations  between the public and commercial sectors.  **E**. Health authorities should develop strategies to improve donor  recruitment and procurement, including establishing organ  procurement organizations, increasing public awareness about organ donation, engaging medical staff, and  involving well-trained coordinators in intensive care unit efforts. The  initiative involves establishing an organ donor foundation, implementing  educational campaigns, establishing community networks, and developing  living donor transplant  programs. Address issues of mistrusts by developing guidelines for organ  donation, establish transparent regulatory oversight, and maintain  good communication between medical  professionals and patients/families.  **F.** The proposed legislation aims to define brain death, establish organ donation and sharing legislation,  establish national donor identification and procurement systems, establish donor and recipient registries, and  promote international cooperation. |

CKD=chronic kidney disease, ESKD=End Stage Kidney Disease, KT=kidney Transplantation,

PKT=Paediatric KT, KRT=Kidney Replacement Therapy, AFRAN=African Association of Nephrology,

ISN=International Society of Nephrology, TTS= The Transplantation Society, IPNA= International

Paediatric Nephrology Association, ISPD= International Society of Peritoneal Dialysis, IPTA=

International Pediatric Transplant Association (IPTA), HIC= high income countries, LMICs= low-and

middle-income countries, LIC=low-income countries, UHC=universal health coverage, LLMIC=low-and

low-middle income countries, NAN= Nigerian Association of Nephrology, PNAN= Paediatric Nephrology

Association of Nigeria, TAN= Transplant Association of Nigeria, MMF=mycophenolate mofetil, CNIs=

calcineurin inhibitors, LDKT=living donation kidney transplantation, DDKT=deceased donation kidney

transplantation, HLA=human leucocyte antigen, WHO=World Health Organization, LTFU=loss to follow

up, PD=peritoneal dialysis, HD=haemodialysis, GKH= Global Kidney Health, WHA=World Health

Assembly
