# Supplementary material for: Inequitable paediatric kidney transplantation in resource-limited countries: expert recommendations for Nigeria – a scoping review
Source: BMJ Glob Health. 2025 Dec 5;10(12):e017023. doi: 10.1136/bmjgh-2024-017023 (PMC12684210; doi:10.1136/bmjgh-2024-017023)
Supplement: online supplemental file 4 [file bmjgh-10-12-s004.docx]

**Supplemental Table 4. Challenges and solutions for expanding paediatric kidney transplantation in resource-limited countries.** ^78^

| **Challenges** | **Solutions** |
| --- | --- |
| **Inadequate government-backed public funding.** ^21, 22, 25, 26, 29, 42, 44-46, 48-50, 52, 53, 59, 60, 62-64, 69-74^ | |
| **A**.OOP payments for high costs of donor investigations, transplant surgeries, and follow-up care, combined with limited government support and insurance coverage, lead to catastrophic health expenditures and increased poverty.  **B.** High costs of inductive immunosuppressants, long-term IST and blood-levels monitoring of immunosuppressants are unsustainable and often lead to allograft rejection when missed/discontinued. | **A**. KT financing should move from OOP payments to social insurance schemes and/or government payment for all or parts of the KT process and/or government-community partnerships (The KT process includes investigations, surgeries, IST, follow-up care, payment for loss of workhours, travels, and accommodation fees for donors, and follow-up of care of donors and recipients).  Other sources of KT financing to supplement government resources include philanthropic organizations, crowdfunding, financial aid programmes, donor agencies and international partnerships.  **B**. Partnership with pharmaceutical companies for manufacturing or importing affordable immunosuppressants or low-cost generic medications. |
| **Inadequate infrastructural and transplant workforce support.** ^21, 22, 24-26, 29, 42, 44, 45, 47-49, 51-53, 56, 58-64, 66-74^ | |
| **A**.There is inadequate laboratory support, including insufficient tissue typing, cross-matching, and immunosuppressant monitoring. The capacity for deceased donations is limited.  **B**.There is a shortage of transplant specialists, such as nephrologists and transplant surgeons.  **C**. Access to tertiary-level healthcare is restricted, and there are no programs for paediatric haemodialysis.  **D**. A renal registry and wait-list are inadequate, and there are also significant technological and logistical resource shortages. | **A/C/D**. Expand and upgrade KT centres to meet international standards equipped with necessary laboratory, radiological, investigative equipment. Ensuring effective operations through technical support. Engage with governmental and non-governmental organizations, and foster public-private partnerships. Seamless transition from adult KT to paediatric KT resources.  **B.**Form alliances with national and international transplant centres for training and support. Task shifting and sharing among allied health professionals, including nurses, pharmacists, and community health workers, to take up uncomplicated tasks. Implement specialized training programs for healthcare professionals in KT/PKT and collaborate with academic institutions for knowledge exchange and innovation**.** Develop training modules, protocols for diagnosis, and KT or referral. |
| **Shortages of living and deceased organs.** ^22, 24, 29, 43, 45-56, 58-63, 66-74^ | |
| **A**.Most organ donations are living-related, with female donors outnumbering male recipients. Age differences between donors and recipients can hinder kidney pair donations due to concerns about allograft outcomes.  The shortage of organ pools, both living-related and unrelated, stems from ignorance, cultural superstitions, and socio-religious beliefs. The increasing burden of CKD and the high failure rates of transplants exacerbate this issue.  Misunderstandings about post-donation care and a lack of public awareness about organ donation further limit the donor pool. Mistrust of medical professionals and cultural beliefs complicate the transplantation process.  **B**. DDKT is less common than LDKT, due in part to a lack of legislation and clear brain death diagnoses. The infrastructure for deceased donation is underdeveloped, with insufficient organizations to coordinate procurement and inadequate communication regarding potential donors. | **A.** Health authorities should promote living donation by developing workable programmes to enhance organ donation through the following strategies:  -To increase public awareness, implement nationwide awareness campaigns to correct myths about organ donation. Conduct comprehensive public awareness activities, including statewide campaigns and educational programs.  -Integrate KT education into school curricula.  -Partner with community leaders and religious organizations to promote organ donation.  -Launch campaigns: Reduce refusal rates through public education.  -Target outreach: Conduct initiatives in community spaces and schools with support from religious leaders.  - Build networks: Use social media to connect patients and families.  -Standardize procedures: Create consistent organ procurement protocols.  -Establish renal registries: Set up donor and recipient registries for fair allocation.  - Implement oversight: Ensure safety and prevent unethical practices.  -Foster communication: Maintain open communication between professionals and families.  **B**. Increase deceased donations via the following strategies:  - Develop or have a clear legal framework.  -Define brain death by having legislation on brain death and organ donation.  -Create national systems by developing donor identification and procurement systems.  -Ensure fair allocation by having organizations overseeing donor and recipient registries.  -Encourage collaboration with governments for effective organ donation laws.  - Raise awareness among intensivists and emergency staff on donor detection.  -Engage Intensive Care Unit Staff and train staff to communicate with families of potential deceased donors.  - Involve trained organ donation coordinators. |
| **Inadequate legislative and regulatory framework for living and deceased KT.** ^21, 22, 24-26, 47, 48, 50, 52, 53, 58-64, 66, 67-74^ | |
| **A**.Lack of strong regulations and limited laws on organ donation and transplantation restrict access to LDKT and DDKT and worsen unethical practices and commercialization of donation | **A.**It is essential to establish national legislation and transparent organ allocation systems for LDKT and DDKT. Enforce laws that will safeguard the rights of all parties involved in KT. Adopting and enforcing international standards help ensure ethical practices. Implement penalties for violations of transplant regulations to deter unethical practices and promote integrity in the system. |
| **Uneven distribution of KT facilities and renal care services.** ^22, 26, 46, 47, 52, 53, 60, 66, 69, 74^ | |
| **A**.Rural areas in Africa lack specialized healthcare facilities, making transplantation services inaccessible due to high transportation costs. Nearly 50% of the population lives in these regions, increasing healthcare disparities. Weak referral systems and unreliable transportation further limit access to paediatric kidney care. | **A**.Establish new KT centres in underserved areas and upgrade existing facilities. Allocate government funding to support renal transplantation programs. Replicate successful transplant partnerships from other regions to underserved areas. Create and maintain KT registries to monitor patient outcomes and enhance access to services**.** |

KT=kidney transplantation, DDKT=deceased donation kidney transplantation, LDKT=living donation kidney transplantation, CKD=chronic kidney disease, IST=immunosuppressive therapy, OOP=out-of-pocket
