# Supplementary material for: Inequitable paediatric kidney transplantation in resource-limited countries: expert recommendations for Nigeria – a scoping review
Source: BMJ Glob Health. 2025 Dec 5;10(12):e017023. doi: 10.1136/bmjgh-2024-017023 (PMC12684210; doi:10.1136/bmjgh-2024-017023)
Supplement: online supplemental file 5 [file bmjgh-10-12-s005.docx]

**Supplemental Table 5. A consensus for expanding paediatric kidney transplantation in Nigeria via questionnaire analysis**

| Solution/Action plan | Basis | Efficiency | Equity | Good quality of care | Avoid catastrophic health expenditure | Sustainability | Consensus agreement to all the 5 indicators |
| --- | --- | --- | --- | --- | --- | --- | --- |
| 1. Generate momentum to  form Nigeria’s Transplant  Community (NTC) | The NTC will  serve as the  advocacy pressure  group. The NTC  will collaborate  internationally  with ISN, TTS,  IPNA, ISPD,  IPTA, and  AFRAN to  develop a “Sister  Transplant  Centre | 92.9% | 100% | 92.9% | 85.7% | 100% | Yes (85.7%) |
| 2. The Paediatric Renal  Registry (already  launched in September  2019). 34 | The renal registry  provides  documents to be  used by the NTC  for monitoring trends and for lobbying and  advocacy for  PKT policymaking | 100% | 92.9% | 85.7% | 85.7% | 92.9% | Yes (85.7%) |
| 3(a). Provide ethical and  regulatory legislation for  living donation. | To prevent organ  trafficking and  improve access to  KT, ethical  regulations must  be established to  ensure  transparency in  organ donation,  allocation, and  transplantation | 100% | 100% | 100% | 78.5% | 100% | Yes (78.5%) |
| 3(b). Provide ethical and  regulatory legislation regarding age of a living donor. | The existing  Act  allows living  donation of tissue  if the donor who is  18 years and  above gives  informed consent. | 85.7% | 100% | 100% | 92.9% | 92.9% | Yes (85.7%) |
| 3 (c). Provide ethical and  regulatory legislation against commodification of human organs and tissues. | The existing Act  prohibits the  harvest of organs  and tissue from  living donors for  commercial  reasons and  benefits,  including sale or  merchandise | 78.5% | 92.9% | 85.7% | 78.5% | 85.7% | Yes (78.5%) |
| 3 (d). Provide ethical and  regulatory legislation for health care facilities that can provide KT services. | The existing Act  requires that  tissue removal  from a living  donor for  transplantation  must take place in  an authorized  hospital under the  written authority  of a medical  practitioner who  is not the lead  participant in the  transplant. | 92.9% | 92.9% | 100% | 78.5% | 92.9% | Yes (78.5%) |
| 3 (e). Provide ethical and  regulatory legislation prohibiting financial inducements for  living donation. | The existing Act  prohibits donors  from receiving  any financial or  other reward,  except for  reasonable  reimbursement of  costs incurred in  providing the  donation. | 92.9% | 100% | 92.9% | 78.5% | 85.7% | Yes (78.5%) |
| 3 (f). Provide ethical and  regulatory legislation regarding acceptable payments for expenses incurred by living donors during the process of donation. | The existing Act  stipulates that  tissue cannot be  sold or traded,  except for  inevitable  expenses incurred  during organ  donations in  licensed health  institutions | 100% | 100% | 100% | 100% | 100% | Yes (100%) |
| 3 (g). The NTC should  lobby the Significant  Stakeholders to amend the legislation to allow informing the living  donors of the risks of  transplant surgery | Living donors  must know the  peri-operative and  long-term risks of  nephrectomy | 100% | 100% | 100% | 92.9% | 100% | Yes (92.9%) |
| 3 (h). The NTC should  lobby the Significant  Stakeholders to amend the legislation to make compulsory the follow-up on living  donors | Follow-up of  donors should be  made compulsory  for lifetime  monitoring of  evolving complications of  organ donation | 92.9% | 92.9% | 92.9% | 78.5% | 92.9% | Yes (78.5%) |
| 3 (i). The NTC should  lobby the Significant  Stakeholders to amend the legislation to allow compulsory  care for donors that may  have unexpected adverse  effects following  transplant nephrectomy | Providing  economic and  social security for  donors should be  an essential part of  comprehensive  care for  CKD/ESKD,  which will  increase the  willingness of  potential donors  to consider organ  donation | 92.9% | 92.9% | 85.7% | 85.7% | 85.7% | Yes (85.7%) |
| 3 (j). The NTC should  establish an Organ Donor  Foundation to increase the  living donor pool via  expanded donor criteria  and transplants between  blood-group-incompatible pairs. | This foundation  can work towards  promoting a  culture of organ  donation in  Nigeria and  addressing any  misconceptions  surrounding  transplantation. | 78.5% | 85.7% | 78.5% | 92.9% | 85.7% | Yes (78.5%) |
| 3 (k). The NTC should  establish an Organ Donor  Foundation to increase the  living donor pool via  paired exchanges of  kidneys | This foundation  can work towards  promoting a  culture of organ  donation and  addressing any  misconceptions  surrounding  transplantation | 100% | 85.7% | 92.9% | 92.9% | 85.7% | Yes (85.7%) |
| 3 (l). Intensive public  enlightenment campaigns  to clarify cultural and  religious biases against  living donation. | The NTC shall  engage in  intensive public  enlightenment  campaigns to  promote safe and  responsible organ  donation. | 100% | 100% | 100% | 92.9% | 100% | Yes (92.9%) |
| 4 (a). Provide legal,  ethical, and regulatory  frameworks for deceased  donation. | The existing Act  permits  competent  persons to donate  their body or  tissue after death  via written  statements or  wills. | 92.9% | 92.9% | 100% | 85.7% | 92.9% | Yes (85.7%) |
| 4 (b). Donors should have the rights to choose the health facility and/or persons that will be recipients of their donations. | The existing Act  states that the  donor may  designate a health  establishment or a  person as the  recipient of their donations. | 100% | 100% | 100% | 92.9% | 100% | Yes (92.9%) |
| 4 (c). Organs from deceased persons can be transplanted to living persons. | The existing Act  stipulates that  organs donated  from a deceased  person can be  used for  transplantation in  a living person | 100% | 92.9% | 100% | 92.9% | 92.9% | Yes (92.9%) |
| 4 (d). Allow a donor the right to withdraw donation before the harvest of an organ. | The existing Act stipulates that a donor may  withdraw  donation before  the removal of the  organ for  transplantation.  The revocation  process is the  same as the  donation  process. | 92.9% | 92.9% | 100% | 100% | 92.9% | Yes (92.9%) |
| 4 (e). The NTC should  lobby significant  stakeholders to amend the legislation to provide a definition for  brain-dead person. ^88^ | Brain death  definition must be  clear and  unambiguous.  The medical team  that certifies a  brain-dead person  must be different  from the  harvesting team,  transplant team,  and Organ  Procurements  Organizations, or  Organ  Procurement and  Transplantation  Networks. | 100% | 100% | 100% | 100% | 100% | Yes (100%) |
| 4 (f). The NTC should  lobby Significant  Stakeholders to amend the legislation  to make deceased  donation possible for  persons who have enrolled  in an opt-in donation  program before death | This will increase  the pool of  deceased organs  available for  transplantation | 92.9% | 92.9% | 100% | 92.9% | 92.9% | Yes (92.9%) |
| 4 (g). The NTC should  lobby Significant  Stakeholders to amend the legislation  to make deceased  donation possible after  death by obtaining harvest  consent from deceased  families or next-of-kin. | This will increase  the pool of  deceased organs  available for  transplantation. | 92.9% | 78.5% | 85.7% | 92.9% | 92.9% | Yes (78.5%) |
| 4 (h). The NTC should  lobby Significant  Stakeholders to amend the legislation to recognize the noble act of donors during funeral services,  bereavement counselling,  and memorial events as  follow-up of deceased  donation. Lobby to  legislate for a posthumous  national award for such  deceased donor | This will  encourage more  people to want to  be a deceased  donor | 92.9% | 92.9% | 100% | 100% | 100% | Yes (92.9%) |
| 4 (i). The NTC should  lobby Significant  Stakeholders to amend the legislation to make a case for  paediatric priority in  deceased-donor organ  allocation. | Prioritizing  children for organ  transplantation  will not affect  adult transplant  waiting times, and  both adult and children will  ultimately benefit  from  transplantation | 92.9% | 85.7% | 85.7% | 92.9% | 92.9% | Yes (85.7%) |
| 4 (j). The NTC shall help  to establish an Organ  Procurement  Organization (OPO) in  each of the 6 proposed  PKT Centre. The OPO will have access to organs  of brain-dead persons in their respective geopolitical regions of the  country | The OPO will  ethically harvest  and transport  organs for  intended  recipients in the six  PKT centres.  They will use air  ambulances,  helicopters, and  commercial  airplanes as  needed to ensure  safe and efficient  transportation and  will be  responsible for  perfusing and  preserving the  organs in optimal  conditions | 92.9% | 100% | 100% | 100% | 92.9% | 92.9% |
| 4 (k). The NTC shall help  to establish an Organ  Allocation Committee  (OAC) in each of the six  proposed PKT centres. The  OACs shall receive tissue  samples from the OPOs. | The OAC will  establish the  NCAS to quickly  identify the best  match organ for  intended  recipients. The  result will be  forwarded to the  OPO in each of  the six regions | 100% | 92.9% | 100% | 100% | 85.7% | Yes (85.7%) |
| 5 (a). Overall, optimize  public funding and  financing for an expanded  PKT | To ensure access  to quality kidney  care for all, we  must advocate for  better funding for  KT programs and  eliminate  catastrophic OOP  health  expenditures for CKD care and  KT | 85.7% | 78.5% | 92.9% | 92.9% | 92.9% | Yes (78.5%) |
| 5 (b). Lobby the significant stakeholders to make amendment that will allow CKD/CKD-5 children to benefit from the Basic  Health Care Provision  Fund (BHCPF) | The  BHCPF is an  important step  towards achieving  universal health  coverage and  equitable access  to healthcare, including the benefits to CKD/CKD-5 children. | 92.9% | 92.9% | 100% | 92.9% | 92.9% | Yes (92.9%) |
| 5 (c). The  NTC should lobby that adults and children with  CKD/CKD-5 be  categorized as vulnerable  and should enjoy the Vulnerable Group Fund under the BHCPF. | Nigeria has  passed a new law  that makes health  insurance  compulsory for all  citizens and legal  residents. The law  establishes the  Vulnerable Group  Fund (VGF) to  Support vulnerable groups  such as children  under five,  pregnant women,  older people,  individuals with  disabilities, and  the poor. | 92.9% | 92.9% | 85.7% | 92.9% | 85.7% | Yes (85.7%) |
| 5 (d). The NTC  should lobby for the  BHCPF to be expanded to  fund adult and paediatric  KT services at the levels  of the public tertiary  teaching hospitals, under  the administration of the  federal government,  through the NHIA  Gateway | This will make the  chronic care of  CKD/CKD-5  (Preventive,  conservative,  dialysis, and KT)  free of cost as part  of UHC access.  Adults and  children with  CKD/CKD-5  should be  classified as part  of the Vulnerable  Group | 85.7% | 92.9% | 92.9% | 85.7% | 92.9% | Yes (85.7%) |
| 5 (e). Secondly, the NTC  should lobby Significant  Stakeholders to enact a  law that will enable a standalone funding  pathway for the total package of renal care to be  called the National Renal Care Fund. ^86^ | To enhance renal  care, measures  include  establishing care  centres,  preventive  nephrology,  funding,  monitoring, and  evaluation,  developing a  National Kidney  Institute, and  fostering strong  relationships. The  Nigerian  Association of  Nephrology  initially proposed  these  improvements | 85.7% | 85.7% | 92.9% | 100% | 92.9% | 85.7% |
| 5 (f). The  NTC must advocate for  the expansion of fiscal  space. This can be done by  campaigning for an annual  financial allocation from  the Federal Government  of Nigeria of not less than  15% of the Consolidated  Revenue Fund (CRF) to fund health. ^28^ | This will make  funds available  for the NHIA  Gateway and the  proposed National  Renal Care Fund. | 85.7% | 100% | 92.9% | 100% | 85.7% | Yes (85.7%) |
| 5 (g). The NTC should  lobby the Significant  Stakeholders to increase  national fiscal space for  national funding for  health. Companies can be  subjected to special CKD  taxation through  profits and capital gains as  part of their corporate  social responsibilities. ^28^ | This will make  funds available  for the NHIA  Gateway and the  proposed National  Renal Care Fund. | 92.9% | 100% | 100% | 92.9% | 85.7% | 85.7% |
| 5 (h). The NTC should  lobby the Significant  Stakeholders to increase  national health funding,  commercial services such  as mobile phone use,  financial transactions, and  air travel can be subject to  special CKD taxation. ^28^ | This will make funds available  for the NHIA  Gateway and the  proposed National  Renal Care Fund | 92.9% | 100% | 85.7% | 100% | 92.9% | Yes (85.7%) |
| 5 (i). The NTC should also  lobby the Significant  Stakeholders to remove  inequitable subsidies by  freeing up fiscal space for  health funding. For  instance, removing  petroleum subsidies can  redirect funds towards  health). ^28^ | This will make  funds available  for the NHIA  Gateway and the  proposed National  Renal Care Fund | 92.9% | 78.5% | 100% | 100% | 85.7% | Yes (78.5%) |
| 5 (j). The NTC through  public campaigns, should  encourage private funding  via communal non-  governmental  philanthropic fund  schemes and crowd-  funding | This will make  funds available  for the NHIA  Gateway and the  proposed National  Renal Care Fund | 92.9% | 100% | 92.9% | 100% | 85.7% | 85.7% |
| 5 (k). The NTC can  recommend a cheaper and  equally effective  azathioprine instead of  MMF as maintenance  immunosuppressive  therapy | This is a cost  saving measure  that will make  residual funds  available for more  patients with  CKD-5 to access  KT | 85.7% | 71.4% | 71.4% | 92.9% | 92.9% | Yes (71.4%) |
| 5 (l). The NTC can use  generic CNIs as  maintenance  immunosuppressive  therapy | This is a cost  saving measure  that will make  residual funds  available for more  patients with CKD-5 to access  KT | 85.7% | 78.5% | 78.5% | 92.9% | 85.7% | Yes (78.5%) |
| 5 (m). The NTC can  recommend the reduction  of the dose of CNIs  needed by combining with  cheaper and safe  ketoconazole, a metabolic  inhibitor | This is a cost  saving measure  that will make  residual funds  available for more  patients with  CKD-5 to access  KT | 92.9% | 85.7% | 92.9% | 92.9% | 92.9% | Yes (85.7%) |
| 5 (n). The NTC should  advocate for the inclusion  of immunosuppressant  drugs as essential  medicines for Nigeria | This is a cost  saving measure  that will make  residual funds  available for more  patients with  CKD-5 to access KT | 92.9% | 92.9% | 92.9% | 92.9% | 92.9% | Yes (92.9%) |
| 5 (o). The NTC should  encourage a more  sustainable chronic  haemodialysis  programme (in-country  preparation of dialysis  fluid) | This is a cost  saving measure  that will make  residual funds  available for more  patients with  CKD-5 to access  KT | 100% | 100% | 92.9% | 100% | 100% | Yes (92.9%) |
| 5 (p). The NTC should  encourage a more  sustainable chronic  haemodialysis  programme (Re-use of  dialyzers) | This is a cost  saving measure  that will make  residual funds  available for more  patients with  CKD-5 to access  KT | 71.4% | 92.9% | 64.3% | 92.9% | 85.7% | Yes (64.3%) |
| 5 (q). The NTC should  encourage a more  sustainable chronic  haemodialysis  programme (Use of basic  haemodialysis machines) | This is a cost  saving measure  that will make  residual funds  available for more  patients with  CKD-5 to access  KT | 78.5% | 92.9% | 78.5% | 85.7% | 92.9% | Yes (78.5%) |
| 5 (r). The NTC should  encourage a more  sustainable chronic  haemodialysis  programme (Pre-emptive  AV-fistula construction) | This is a cost  saving measure  that will make  residual funds  available for more  patients with  CKD-5 to access  KT | 100% | 85.7% | 92.9% | 92.9% | 64.3% | Yes (64.3%) |
| 6 (a). Build Health  workforce | Skilled  workforce,  including  transplant  surgeons,  nephrologists,  anaesthetists,  intensivists,  nurses,  pharmacists,  histopathologists,  social workers,  psychologists, and psychiatrists is  required to build a  renal transplant  workforce | 100% | 100% | 100% | 92.9% | 92.9% | Yes (92.9%) |
| 6 (b). The NTC can  collaborate with AFRAN,  IPNA, IPTA, TTS and  ISN to continue with 1– 2-  year fellowship training  programs targeted specifically at KT Science | This will build  health workforce  expertise  necessary for  maintaining and  expanding PKT  Services. | 100% | 92.9% | 100% | 92.9% | 92.9% | Yes (92.9%) |
| 6 (c). The NTC should  also advocate with  Nigeria Post-graduate  Medical College and the  West African College of  Physicians (Paediatrics) to  make PKT Science  mandatory in  curriculum for  postdoctoral training | This will build  health workforce  expertise  necessary for  maintaining and  expanding PKT  Services. | 100% | 100% | 100% | 100% | 100% | Yes (100%) |
| 6 (d). Task shifting and  sharing among allied  health professionals,  including nurses,  pharmacists, and  community health  workers, to take up  uncomplicated  tasks. | This will reduce  the burden of  work on the few  available  paediatric  nephrologists  providing care for  KT donors and  recipients. | 64.3% | 71.4% | 71.4% | 85.7% | 71.4% | No (64.3%) |
| 6 (e). Develop training  modules, protocols for  diagnosis, and treatment  or referral. | This will build  health workforce  expertise  necessary for  maintaining and  expanding PKT  Services | 100% | 100% | 100% | 92.9% | 100% | 92.9% |
| 7 (a). Build healthcare  infrastructure at PKT  centres | Six  government-owned  PKT centres  should be  established in  each of the 6  geopolitical  regions of the  country | 100% | 100% | 100% | 92.9% | 92.9% | Yes (92.9%) |
| 7 (b). It is advisable to  prioritize living-donor  programs first. Once the  appropriate infrastructure  is in place, then the  deceased donation  program can be  considered. | This approach  ensures that the  transplant  program starts off  with a sustainable  option and can  gradually expand  to include  deceased donation  when the  necessary  resources are  available. | 100% | 92.9% | 92.9% | 92.9% | 100% | Yes (92.9%) |

RRT=renal replacement therapy, PAR percentage agreement response, PNAN=Paediatric Nephrology

Association of Nigeria, TAN=Transplant Association of Nigeria, NAN=Nigerian Association of

Nephrology, NGOs=non-governmental organizations, NHIA=National Health Insurance Authority Act, 2022, Significant Stakeholders (National/States Legislative Assemblies, the Honourable Minister of Health, the Federal Ministry of Health, the National Council on Health, Technical Committee of the National Council on Health, and the National Tertiary Health Institutions Standard Committee), CAN=Christian Association of Nigeria, NSCIA= Nigerian

Supreme Councils for Islamic Affairs, ISN=International Society of Nigeria, TTS=The Transplant Society,

IPNA=International Paediatric Nephrology Association, ISPD= International Society of Peritoneal

Dialysis, IPTA=International Paediatric Transplant Association, AFRAN= African Association of

Nephrology, CNI=Calcineurin inhibitors, MMF=Mycophenolate Mofetil, NHAct= National Health Act

2014, NCAS=National Computer Allocation System, UHC=universal health coverage.

**Efficiency**: Healthcare efficiency measures the relationship between resource inputs and health outcomes

to achieve health system goals and obtain the best value for money. 48

**Equity:** Equity refers to the absence of unfair, avoidable, or remediable differences among groups of

people based on various dimensions of inequality, including sex, gender, ethnicity, disability, or sexual

orientation. 49

**Quality of care**: A good quality of care is characterized by being timely, effective, equitable, safe, and

people centred. It should be evidence-based, avoiding harm to those who receive it, and responsive to

individual preferences, needs, and values. 50

**Catastrophic health expenditure**: When health spending surpasses 40% of a household's non-subsistence

income, it becomes catastrophic and can result in poorer treatment outcomes and reduced use of health

services. 51, 52

**Sustainability**: WHO defines a Sustainable Healthcare System as one that improves health while

minimizing negative impacts on the environment and leveraging opportunities for future generations'

benefit.53
