# Supplementary material for: Inequitable paediatric kidney transplantation in resource-limited countries: expert recommendations for Nigeria – a scoping review
Source: BMJ Glob Health. 2025 Dec 5;10(12):e017023. doi: 10.1136/bmjgh-2024-017023 (PMC12684210; doi:10.1136/bmjgh-2024-017023)
Supplement: online supplemental file 6 [file bmjgh-10-12-s006.docx]

**Supplemental Table 6. The Preferred Reporting Items for Systematic reviews and Meta-Analyses extension for Scoping Reviews (PRISMA-ScR) Checklist for “A scoping review on equitable paediatric kidney transplantation in resource-limited countries: Expert recommendations for Nigeria.”**

| **SECTION** | **ITEM** | **PRISMA-ScR CHECKLIST ITEM** | **REPORTED ON PAGE #** |
| --- | --- | --- | --- |
| **TITLE** | | | |
| Title | 1 | Identify the report as a scoping review. | Now visible in the new Title (Page 1/lines 1-2) |
| **ABSTRACT** | | | |
| Structured summary | 2 | Provide a structured summary that includes (as applicable): background, objectives, eligibility criteria, sources of evidence, charting methods, results, and conclusions that relate to the review questions and objectives. | Find this under Abstract on Page 3-4/lines 43-73 |
| **INTRODUCTION** | | | |
| Rationale | 3 | Describe the rationale for the review in the context of what is already known. Explain why the review questions/objectives lend themselves to a scoping review approach. | Context of what is already known can be found on Pages 6-8. The scoping review becomes inevitable because it is important to synthesise various strategies that have helped to establish PKT in resource limited countries. |
| Objectives | 4 | Provide an explicit statement of the questions and objectives being addressed with reference to their key elements (e.g., population or participants, concepts, and context) or other relevant key elements used to conceptualize the review questions and/or objectives. | The specific objective of the scoping review was clearly stated on Page 8, lines 163-165. |
| **METHODS** | | | |
| Protocol and registration | 5 | Indicate whether a review protocol exists; state if and where it can be accessed (e.g., a Web address); and if available, provide registration information, including the registration number. | The detail explanation of the review’s Protocol can be found on Page 9, lines 182-186. |
| Eligibility criteria | 6 | Specify characteristics of the sources of evidence used as eligibility criteria (e.g., years considered, language, and publication status), and provide a rationale. | The eligibility criteria were clearly stated on Page 10, lines 196-198. |
| Information sources* | 7 | Describe all information sources in the search (e.g., databases with dates of coverage and contact with authors to identify additional sources), as well as the date the most recent search was executed. | Information sources can be found on Page 9, lines 188 to 189. |
| Search | 8 | Present the full electronic search strategy for at least 1 database, including any limits used, such that it could be repeated. | The full electronic search for two of three databases can be in Supplemental **Table 1.** |
| Selection of sources of evidence† | 9 | State the process for selecting sources of evidence (i.e., screening and eligibility) included in the scoping review. | The screening and eligibility for selecting sources of evidence can also be found in Pages 9 &10, lines 188-194 |
| Data charting process‡ | 10 | Describe the methods of charting data from the included sources of evidence (e.g., calibrated forms or forms that have been tested by the team before their use, and whether data charting was done independently or in duplicate) and any processes for obtaining and confirming data from investigators. | The methods of charting data from the included sources of evidence can be seen on Page 10, lines 194-204 and in **Supplemental Tables 2&3** |
| Data items | 11 | List and define all variables for which data were sought and any assumptions and simplifications made. | Find the details of all the variables sought in **Supplemental Tables 2, 3 &5**. |
| Critical appraisal of individual sources of evidence§ | 12 | If done, provide a rationale for conducting a critical appraisal of included sources of evidence; describe the methods used and how this information was used in any data synthesis (if appropriate). | Statement on critical appraisal can be seen on Pages 12-13, lines 242-277. Also on Pages 14-15, lines 290-328.**Supplemental Tables 2, 3 &5**. |
| Synthesis of results | 13 | Describe the methods of handling and summarizing the data that were charted. | Also inferred on Pages 10, lines 205-206. |
| **RESULTS** | | | |
| Selection of sources of evidence | 14 | Give numbers of sources of evidence screened, assessed for eligibility, and included in the review, with reasons for exclusions at each stage, ideally using a flow diagram. | Summarise in the PRISMA flow chart (**Figure 2**). |
| Characteristics of sources of evidence | 15 | For each source of evidence, present characteristics for which data were charted and provide the citations. | Also find this in the PRISMA flow chart (**Figure 2**). |
| Critical appraisal within sources of evidence | 16 | If done, present data on critical appraisal of included sources of evidence (see item 12). | Also find this in the PRISMA flow chart (**Figure 2**) |
| Results of individual sources of evidence | 17 | For each included source of evidence, present the relevant data that were charted that relate to the review questions and objectives. | Also find this in the PRISMA flow chart (**Figure 2**) |
| Synthesis of results | 18 | Summarize and/or present the charting results as they relate to the review questions and objectives. | This has been summarised in **Supplemental Table 5**. |
| **DISCUSSION** | | | |
| Summary of evidence | 19 | Summarize the main results (including an overview of concepts, themes, and types of evidence available), link to the review questions and objectives, and consider the relevance to key groups. | Find the summary of the main results in Result section, Pages 12-15. |
| Limitations | 20 | Discuss the limitations of the scoping review process. | Find limitations of study on Pages 21-22, lines 488-493. |
| Conclusions | 21 | Provide a general interpretation of the results with respect to the review questions and objectives, as well as potential implications and/or next steps. | Find conclusions on Page 22, lines 504-509. |
| **FUNDING** | | | |
| Funding | 22 | Describe sources of funding for the included sources of evidence, as well as sources of funding for the scoping review. Describe the role of the funders of the scoping review. | Find statement on funding on Page 23, lines 523-524. |

JBI = Joanna Briggs Institute; PRISMA-ScR = Preferred Reporting Items for Systematic reviews and Meta-Analyses extension for Scoping Reviews.

* Where *sources of evidence* (see second footnote) are compiled from, such as bibliographic databases, social media platforms, and Web sites.

† A more inclusive/heterogeneous term used to account for the different types of evidence or data sources (e.g., quantitative and/or qualitative research, expert opinion, and policy documents) that may be eligible in a scoping review as opposed to only studies. This is not to be confused with *information sources* (see first footnote).

‡ The frameworks by Arksey and O’Malley (6) and Levac and colleagues (7) and the JBI guidance (4, 5) refer to the process of data extraction in a scoping review as data charting*.*

§ The process of systematically examining research evidence to assess its validity, results, and relevance before using it to inform a decision. This term is used for items 12 and 19 instead of "risk of bias" (which is more applicable to systematic reviews of interventions) to include and acknowledge the various sources of evidence that may be used in a scoping review (e.g., quantitative and/or qualitative research, expert opinion, and policy document).

*From:* Tricco AC, Lillie E, Zarin W, O'Brien KK, Colquhoun H, Levac D, et al. PRISMA Extension for Scoping Reviews (PRISMAScR): Checklist and Explanation. Ann Intern Med. 2018;169:467–473. [doi: 10.7326/M18-0850](http://annals.org/aim/fullarticle/2700389/prisma-extension-scoping-reviews-prisma-scr-checklist-explanation).
